# Tuning the tropism and infectivity of SARS-CoV-2 virus-like particles for mRNA delivery

**DOI:** 10.1093/nar/gkaf133

**Published:** 2025-03-04

**Authors:** Qi Yang, Bruce A Davidson, Petar Pajic, Xuyang Chen, Omer Gokcumen, Min Gao, Sriram Neelamegham

**Affiliations:** Chemical & Biological Engineering, State University of New York, Buffalo, NY 14260, United States; Department of Anesthesiology, State University of New York, Buffalo, NY 14203, United States; Veterans Administration Western New York Healthcare System, Buffalo, NY 14215, United States; Department of Biological Sciences, State University of New York, Buffalo, NY 14260, United States; Chemical & Biological Engineering, State University of New York, Buffalo, NY 14260, United States; Department of Biological Sciences, State University of New York, Buffalo, NY 14260, United States; Advanced Materials and Liquid Crystal Institute, Kent State University, Kent, OH 44242, United States; Chemical & Biological Engineering, State University of New York, Buffalo, NY 14260, United States; Biomedical Engineering, State University of New York, Buffalo, NY 14260, United States; Cell, Gene and Tissue Engineering Center, State University of New York, Buffalo, NY 14260, United States; Medicine, State University of New York, Buffalo, NY 14260, United States; Clinical & Translational Research Center, Buffalo, NY 14260, United States

## Abstract

Severe acute respiratory syndrome coronavirus 2 (SARS-CoV-2) virus-like particles (VLPs) are ∼100-nm-sized bioinspired mimetics of the authentic virus. We undertook molecular engineering to optimize the VLP platform for messenger RNA (mRNA) delivery. Cloning the nucleocapsid protein upstream of M-IRES-E resulted in a three-plasmid (3P) VLP system that displayed ∼7-fold higher viral entry efficiency compared with VLPs formed by co-transfection with four plasmids. More than 90% of human ACE2-expressing cells could be transduced using these 3P VLPs. Viral tropism could be programmed by switching glycoproteins from other viral strains, including other betacoronaviruses and the vesicular stomatitis virus G protein. An infectious two-plasmid VLP system was also advanced where one vector carried the viral surface glycoprotein and the second carried the remaining SARS-CoV-2 structural proteins and reporter gene. SARS-CoV-2 VLPs could be engineered to carry up to four transgenes, including functional Cas9 mRNA for genome editing. Gene editing of specific target cell types was feasible by modifying VLP tropism. Successful mRNA delivery to mouse lungs suggests that the SARS-CoV-2 VLPs can overcome natural biological barriers to enable pulmonary gene delivery. Overall, the study describes the advancement of the SARS-CoV-2 VLP platform for robust mRNA delivery both *in vitro* and *in vivo*.

## Graphical Abstract

**Graphical Abstract:**
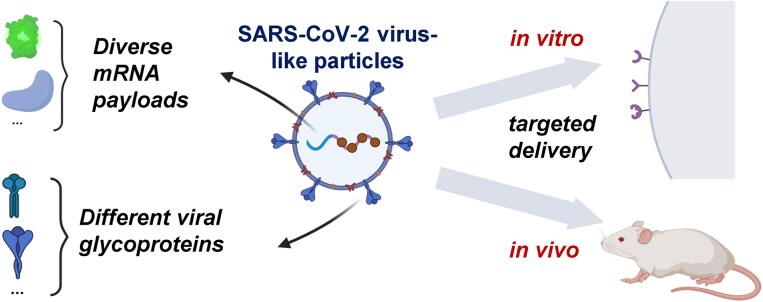

## Introduction

COVID-19 (Coronavirus disease 2019) is caused by the severe acute respiratory syndrome coronavirus 2 (SARS-CoV-2, abbreviated "SARS2"). A number of experimental platforms have been developed to study the biological features of SARS2 and to develop related countermeasures. One of these involves the development of nonreplicative virus-like particles (VLPs) or SARS2 VLPs that resemble the authentic virus as they are self-assembled by co-expression of the four SARS2 structural proteins in cells [1]. These structural proteins include the viral spike (S), nucleocapsid (N), membrane (M), and envelope (E) proteins. VLPs with fewer SARS2 structural proteins may be formulated, but these often result in less infectious agents [2]. SARS2 VLPs are mostly used to advance vaccine development research as these particles are non-replicative, exhibit low toxicity, are more stable than subunit vaccines, and enable the development of immunological response against multiple viral proteins [3–5]. While the earlier SARS2 VLP formulations did not carry a packaging sequence to encapsulate messenger RNA (mRNA) [6–8], Syed *et al.* demonstrated the feasibility of achieving this by including a SARS2 *cis-*acting RNA packaging sequence that engages the SARS2 N protein [9]. The authors showed that tagging a reporter gene to a *cis-*acting segment of the SARS2 genome called “T20”, which encodes for the nonstructural proteins 15 and 16 (nucleotides: 20 080–22 222), enables the formation of a ribonucleoprotein (RNP) complex with N protein that is then packaged into the VLP. In addition to T20, even a smaller segment called “PS9” (nucleotides: 20 080–21 171) allowed similar encapsulation of a reporter gene into the VLP. Such viral particles have been useful in basic science studies that quantify the effect of spike mutations [10] and glycosylation on viral infectivity [11]. The absence of additional viral mRNA in such studies minimizes adverse effects to host cells and ameliorates laboratory safety concerns.

Besides vaccine development and fundamental biomedical investigations, there is growing interest in testing VLPs as a platform for gene delivery to heterologous cells [12, 13]. This is because VLPs are highly customizable, offering distinct advantages over conventional viral and nonviral gene delivery strategies. Unlike 25 nm nonenveloped adeno-associated virus with an ∼5 kb genome, VLPs formulated using SARS2 proteins could potentially carry larger mRNA due to their large 100–120 nm physical size and ∼29.9 kb genome [14, 15]. Such VLPs would be nonintegrative as opposed to lentivirus, and they may potentially result in lower immunogenicity compared with adenovirus that promote T-cell mediated cytotoxicity [16]. In addition, as compared with nonviral delivery strategies like polyplexes, lipoplexes, and lipid nanoparticle formulations which display predilection to liver or spleen [17, 18], the tropism of these viral mimetics may be tuned by modifying their surface glycoproteins. Finally, in systemic circulation, virions like SARS2 exhibit natural organotropism that is quite distinct from synthetic nanoparticles [19]. As examples of recent success, Banskota *et al.* report the engineering of the Friend murine leukemia virus VLPs for efficient base editor targeting or Cas9 RNP delivery *in vitro* and *in vivo* [20]. Segel *et al.* describe a mammalian retrovirus-like protein PEG10 which enables the packaging of cargo mRNA, forming VLPs that can be pseudotyped with vesicular stomatitis virus G protein (VSV-G) or other viral glycoprotein for mRNA delivery into cells [21]. To date, however, to our best knowledge no study has reported the use of SARS2 VLPs to conduct mRNA payload delivery and gene editing.

Here, we present our work to tune the tropism and explore the packaging capacity of VLPs formulated using the SARS2 structural proteins. In one aspect, we performed extensive molecular engineering to reduce the number of plasmids used to synthesize the VLPs from four in the original “four-plasmid” (4P) system [10, 11], to “three-plasmid” (3P) system, and then down to “two-plasmid” (2P) system. To describe the variety of produced particles, VLPs in this manuscript are described using a nomenclature that includes data on the structural proteins, viral glycoprotein, and reporter encapsulated in the VLPs, with Luc-PS9 being the default reporter. For example, 3P SARS2 EGFP-PS9 VLP denotes a 3P VLP containing a SARS2 S glycoprotein and EGFP reporter that is linked to PS9. Our extensive biomolecular engineering resulted in VLPs that are both easier to synthesize and display improved entry properties. *Trans*-complementation with various viral glycoproteins enabled modification of viral tropism. Additionally, mRNA encoding for up to four different proteins could be packaged in a single VLP and gene editing could be enabled by packaging the *Streptococcus pyrogenes* Cas9 mRNA in the particles. Finally, as the pulmonary SARS2 virions can be readily aerosolized [22], we determined that such VLPs may be used for pulmonary gene delivery. Overall, the study describes a streamlined platform for the robust production of SARS2 VLPs, for use in basic science investigations and gene/drug delivery applications.

## Materials and methods

### Biochemicals

All monoclonal antibodies (mAbs) were from mouse unless otherwise stated. These include anti-SARS2 N protein mAb 1035111 (IgG2b, Catalog #: MAB10474), anti-SARS2 spike subunit 2 (S2) mAb 1034617 (IgG2a, Catalog #: MAB10557), Alexa Fluor 647-conjugated anti-human ACE2 mAb 535919 (IgG2a, Catalog #: FAB9332R), and rabbit anti-mouse ACE2 mAb 2818I (IgG, Catalog #: FAB34372G), all from R&D Systems (Minneapolis, MN). Rabbit anti-SARS2 E protein polyclonal antibody (pAb) (Catalog #: 74698), anti-SARS2 M protein mAb E5A8A (IgG1, Catalog #: 15333), HRP-conjugated horse anti-mouse IgG pAb (Catalog #: 7076, RRID: AB_330924), and HRP-conjugated goat anti-rabbit IgG pAb (Catalog #: 7074, RRID: AB_2099233) were purchased from Cell Signaling (Danvers, MA). FITC-conjugated anti-human CD26/DPP4 IgG2a mAb BA5b was purchased from BioLegend (Catalog #: 302704). All other biochemicals were from Sigma Chemicals (St Louis, MO), Gold Biotechnology (Olivette, MO), or ThermoFisher (Waltham, MA) unless otherwise mentioned.

### Cell culture

Human embryonic kidney 293T Lenti-X cells (“293T”) (Catalog #: 632180) were purchased from Clontech/Takara Bio (Mountain View, CA). Stable 293T-human ACE2 (“293T-hACE2”) cells were generously provided by Michael Farzan (Scripps Research, Jupiter, FL). A549 lung carcinoma-overexpressing human ACE2 and TMPRSS2 (“A549-hACE2-TMPRSS2”) cells (Catalog #: a549-hace2tpsa) were purchased from InvivoGen (San Diego, CA). Calu-3 human airway epithelial cells (Catalog #: HTB-55) were from ATCC (Manassas, VA). 293T-DPP4 cells were produced by transducing lentivirus packaged with dipeptidyl peptidase-4 (DPP4) gene into 293T cells. 293T-hACE2-EGFP cells and 293T-EGFP cells were made by transducing lentivirus packaged with EGFP gene into 293T-hACE2 and 293T cells, respectively. Isogenic clones were selected for the 293T-DPP4- and EGFP-bearing cells using fluorescence-activated cell sorting, and these were scaled up for downstream studies. 293T-mouse ACE2 (“293T-mACE2”) cells were produced by transducing lentivirus packaged with mouse ACE2 gene into 293T cells. 293T-hACE2-SLC35A1 single guide RNA (sgRNA) cells were produced by transducing 293T-hACE2 with a pool of two VSV-G pseudotyped lentivirus each carrying an sgRNA targeting *SLC35A1*. These cells were bulk sorted and scaled up prior to use in functional studies. All cells were cultured using Dulbecco’s modified Eagle’s medium (DMEM) supplemented with 10% fetal bovine serum, 1% Antibiotic–Antimycotic, and 1% GlutaMAX supplement. Cultures of Calu-3 were additionally supplemented with 1% nonessential amino acids. 293T-hACE2, 293T-mACE2, 293T-hACE2-SLC35A1 sgRNA, and 293T-DPP4 cultures additionally contained 1 μg/ml puromycin for selection. A549-hACE2-TMPRSS2 culture media contained 100 μg/ml normocin, 0.5 μg/ml puromycin, and 300 μg/ml hygromycin based on manufacturer’s instructions. The whole cell culture was performed in incubators having following characteristics: maintained at 37°C, humidified, and 5% CO_2_ environment.

### Molecular biology

The parent spike plasmid was from our previous work [11]. The original plasmid containing the full-length SARS2 S protein with C-terminal FLAG-tag was generously provided by Michael Farzan [23], and this was then site-directed modified to introduce a D614G mutation. The pcDNA3.1 SARS2 N containing R203M mutation (RRID: Addgene_177952), pcDNA3.1 SARS2 M-IRES-E (RRID:), pcDNA3.1 Luc-PS9 (RRID: Addgene_177942), pcDNA3.1 Luc-T20 (RRID: Addgene_177941), and pcDNA3.1 GFP-PS9 (RRID: Addgene_177944) plasmids were kindly provided by Jennifer Doudna [9]. The pcDNA3.1 N-T2A-M-IRES-E plasmid was made by inserting the N-T2A fragment upstream of M-IRES-E in pcDNA3.1 SARS2 M-IRES-E. The pMD2.G which encodes for VSV-G glycoprotein was a gift from Didier Trono (RRID: Addgene_12259). The pCDNA3.3 Middle East respiratory syndrome (MERS) D12 S plasmid encoding the MERS wild-type (WT) S protein with a 12-amino acid deletion on the C-terminal tail was from David Nemazee (RRID: Addgene_170448). pcDNA3.1 SARS S plasmid encoding the 2002 SARS S protein was kindly provided by Fang Li (RRID: Addgene_145031). The pLEX307-DPP4-puro plasmid encoding the DPP4/CD26 was a gift from Alejandro Chavez and Sho Iketani (RRID: Addgene_158451). The pscALPSpuro-MmACE2 encoding for mouse ACE2 was a gift from Jeremy Luban (RRID: Addgene_158087). The lentiviral dual-promoter (LVDP) vector was kindly provided by Stelios Andreadis (University at Buffalo, Buffalo, NY) [24]. A family of four derivative plasmids was made based on this, including LVDP 2P.1, LVDP 2P.2, LVDP 2P.3, and LVDP 2P.4 each containing different promoter configurations. The LVDP 2P.2.EGFP was produced by replacing the luciferase gene in LVDP 2P.2 with EGFP. The pcDNA3.1 CMV-NME SV40-LucPS9 plasmid was made by replacing the NeoR/KanR gene of pcDNA3.1 SARS2 N-T2A-M-IRES-E with the Luc-PS9 gene cassette. The const.2, const.3, const.4, const.5, and const.6 constructs were produced by successive addition of dTomato, TagBFP, and luciferase reporter genes into the pcDNA3.1 GFP-PS9 plasmid (denoted const.1). These reporter genes were linked using P2A, T2A, or *encephalomyocarditis virus* (EMCV) IRES (internal ribosome entry site) sequences. The pcDNA3.1 GFP-PS9-PS9 plasmid was cloned by inserting a second PS9 packaging sequence to the end of PS9 in pcDNA3.1 GFP-PS9. sgRNA targeting *EGFP*, *SLC35A1*, and human ACE2 (*hACE2*) were cloned into the original pKLV-U6gRNA(BbsI)-PGKpuro2ABFP vector provided by Kosuke Yusa (RRID: Addgene_50946). The pcDNA3.1 Cas9-P2A-dTomato-PS9 and pcDNA3.1 Cas9-P2A-dTomato-T20 plasmids were cloned by replacing the luciferase reporter gene with the Cas9-P2A-dTomato fragment in pcDNA3.1 Luc-PS9 and pcDNA3.1 Luc-T20, respectively. The lentiviral vector pLKO.1 TRC-EGFP was made by replacing the DsRed gene in our previous pLKO.1 TRC-DsRed plasmid with EGFP gene [25]. Constructs were typically produced using the NEB HiFi DNA Assembly kit, and verified using either Sanger and/or Oxford Nanopore whole plasmid sequencing.

### Transfection

293T cells and variants of this cell line were transfected using either the calcium phosphate method [26] or Lipofectamine 2000 reagent following manufacturer’s instructions. Such transfections were used to produce both lentivirus and VLPs, and for the expression of sgRNAs in 293T-hACE2, 293T-hACE2-EGFP, and 293T-EGFP cells. Briefly, cells were plated in either cell culture-treated six-well plates or 150-mm petri dishes 1 day prior to transfection. The next day (day 1), when cell density reached ∼70% confluence, 2 μg DNA was used to transfect each well in a six-well plate, while ∼50 μg DNA was used for each 150-mm petri dish. In all cases, 6–8 h post-transfection, medium was switched to fresh Opti-MEM.

### VLP production

To produce VLPs, 15–20 million 293T cells were seeded in 150-mm petri dishes on day 0, in order to reach ∼70% confluence overnight. In all experiments, ∼50 μg of plasmid encoding for structural proteins was used, unless stated otherwise. Thus, when producing VLPs using the “4P” VLP system, 8.25 μg M-IRES-E, 16.75 μg N, 25 μg Luc-PS9, and 1 μg spike plasmid were co-transfected on day 1. In the case of the “3P” VLP system, cells were co-transfected using 25 μg N-T2A-M-IRES-E, 25 μg Luc-PS9, and 1 μg spike plasmids. Similarly, “2P” VLPs were produced by co-transfecting 293T cells using 50 μg of one of the 2P plasmids, along with 1 μg spike plasmid. In some studies that titrated spike plasmid amounts, spike plasmid fraction was varied while preserving the mass ratio of the remaining constructs encoding for viral structural proteins and Luc/EGFP reporters such that a total of 50 μg plasmid was used for each 150-mm petri dish. In instances where plasmids were linearized prior to transfection for VLP production, 1 μl plasmid DNA (∼2 μg/μl), 2 μl rCutSmart buffer (New England BioLabs, Catalog #: R0532S), 1 μl restriction enzyme, and 16 μl nuclease-free water were added into a 0.2 ml polymerase chain reaction (PCR) tube. The reagents were mixed and incubated at 37°C for 1 h prior to validation of cut by gel electrophoresis. This product was then also used for cell transfection and VLP production. The above “standard” method was used in all studies unless otherwise stated.

In all cases, 6–8 h post-transfection, the cell culture medium was switched to 20 ml fresh Opti-MEM. Then, 48 h thereafter, the cell culture supernatant was harvested, centrifuged at 4000 × *g* for 5 min to remove any floating cells, and filtered through 0.45-μm polyethersulfone (PES) membrane to remove cell debris. In most cases, 20 ml of the clarified supernatant was added into polycarbonate centrifuge bottles (Beckman Coulter, Indianapolis, IN). A 4-inch-long stainless-steel needle was then used to inject 20% (g/ml) sucrose solution at 1/10th sample volume as cushion to the bottom of the tube. Subsequently, the bottles were ultracentrifuged at 150 000 × *g* for 2.5 h at 4°C using a Type 70 Ti rotor in an Optima XE Ultracentrifuge (Beckman Coulter). The opaque VLP pellet obtained was resuspended in 200 μl phosphate-buffered saline (PBS; 1 mM KH_2_PO_4_, 155 mM NaCl, 3 mM Na_2_HPO_4_). When using a larger Type 45 Ti rotor, the above reagent volumes were proportionally scaled. Following this, the VLP sample (100× concentrate) was vortexed thoroughly and spun down in a bench-top centrifuge at 13 000 × *g* for 2 min to remove any remaining debris. The supernatant was transferred into a new microcentrifuge tube. The VLP sample prepared in this manner was either used directly for downstream application or stored at −80°C for future studies. In addition to the ultracentrifugation method above, in one biological repeat we compared the efficacy of 4P versus 3P VLPs by simply concentrating the 20 ml clarified supernatant 100-fold using a PES protein concentrator (100 kDa cutoff, ThermoFisher).

### Pseudovirus production and engineering of stable cell line

VSV-G pseudotyped-lentivirus was produced using the third-generation lentivirus system [27]. 293T producer cells were plated in 150 mm petri dish on day 0 to reach ∼70% confluence the next day. The cells were co-transfected with 18.9 μg pLKO.1 TRC-EGFP, 21.4 μg psPAX2, and 5.8 μg VSV-G plasmids. Then, 6–8 h post-transfection, medium was switched to 20 ml fresh Opti-MEM. The first virus batch was collected at 18–20 h and stored at 4°C. Fresh Opti-MEM containing 10 mM sodium butyrate (Sigma–Aldrich) was then added and second virus batch was collected 16–20 h thereafter. Both batches were pooled and centrifuged at 2000 × *g* for 2 min to remove floating cells, filtered through 0.45-μm PES membrane to remove cell debris, and then ultracentrifuged at 50 000 × *g* for 2 h at 4°C to obtain the viral pellet. The pellet was resuspended in 100 μl Opti-MEM, aliquoted and stored at −80°C for further use.

### Luminescence assay to assess VLPs’ infectivity

The target cells were trypsinized and resuspended at 10^7^ cells/ml. In a 1.5-ml microcentrifuge tube, 50 μl VLP preparation (default volume, unless otherwise specified) was mixed with 80 000 cells (8 μl of stock), along with the addition of 8 μg/ml polybrene. After incubation at room temperature (RT) for 25 min with periodic flicking, the cells were transferred into 96-well plates with additional 150 μl fresh DMEM media. Following overnight culture to allow for reporter expression, the cells were washed with 200 μl PBS buffer and lysed using 50 μl/well cell lysis buffer (Gold Biotechnology) at RT on a shaker for 20 min. In this time, fresh 2× TMCA (Tris-coenzyme A) buffer was made by mixing 20 μl MgCl_2_ (from 500 mM MgCl_2_ 100× stock), 20 μl coenzyme A (from 25 mM 100× stock), 20 μl ATP (from 15 mM 100× stock), 500 μl Tris–HCl (pH = 7.8, from 400 mM 4× stock), and additional 440 μl cell culture water to bring up the volume to 1 ml. Following cell lysis, 50 μl 2× TMCA was added into a 96-well white plate with round bottom followed by 50 μl cell lysate. Then, 1 μl d-luciferin (from 15 mg/ml d-luciferin 100× stock) was added and resulting luminescence was immediately quantified using either a BioTek Synergy4 plate reader (Santa Clara, CA) or a GloMax luminescence microplate reader (Promega, Madison, WI). Studies with mouse tissue were performed identically only using 50 μl mouse lysates produced using tissue lysis buffer (Gold Biotechnology).

### Flow cytometry measurement of receptor expression and VLP entry

Fluorescent reporter expression in VLP-transduced cells was measured using methods identical to the luminescence assay above, only using a BD Fortessa X-20 flow cytometer (San Diego, CA) to quantify fluorescence signal in live cells. The cytometer was also used to quantify receptor expression on various cells. In both cases, cells were trypsinized from tissue culture flasks or six-well plates, and resuspended in HEPES buffer [110 mM NaCl, 10 mM KCl, 2 mM MgCl_2_, 10 mM glucose, 30 mM HEPES (pH = 7.2–7.3)] at 10^7^ cells/ml. Then, 20 μl cells were added into a 1.5-ml microcentrifuge tube along with 0.5–5 μg/ml fluorescent antibodies if necessary for 15–20 min on ice. After washing using HEPES buffer, the cells were resuspended at 2 × 10^6^ cells/ml and analyzed using the cytometer. Mock transduced cells were used as negative control for the VLP entry studies. Both the percentage of cells with more than baseline EGFP signal and mean fluorescence intensity (MFI) of all cells are reported.

### Fluorescence microscopy

A four-well glass chamber slide (Nest Scientific, NJ) was coated with 500 μl of 16 μg/ml matrigel dissolved in PBS for 1 h at 37°C. The slide was washed once with 500 μl PBS and stored at 4°C prior to use. Then, 50 000 293T-hACE2 or A549-hACE2-TMPRSS2 cells were incubated with 50 μl VLP for 25 min at RT before being transferred into the chamber slides along with 500 μl fresh DMEM. Following overnight culture, the cells were washed once with 500 μl PBS and mounted using a few drops of ProLong glass antifade. Fluorescence images were collected using a Zeiss AxioObserver microscope (10×/0.25 NA objective).

### Western blot

VLP samples were denatured in SDS–DTT (sodium dodecyl sulfate-dithiothreitol) blue loading buffer (Cell Signaling) by heating at 98°C for 5–10 min. Then, 10 μl concentrated VLP sample for anti-M and anti-E or 2 μl sample for anti-S2 and anti-N was resolved using a 12% Tris–glycine gel. Following transfer onto a nitrocellulose membrane using a Trans-Blot Turbo Transfer System (Bio-Rad, Hercules, CA), the membranes were blocked for 1 h at RT using TBST (Tris-buffered saline with Tween, 100 mM sodium chloride, 20 mM Tris–HCl, 0.1% Tween-20) containing 5% nonfat milk. Primary antibody was then added at manufacturer recommended concentrations in TBST solution containing 2% nonfat milk at 4°C overnight. The next day, the membrane was washed using TBST four times with each wash lasting 5 min at RT. The membrane was then treated with HRP-conjugated secondary antibody for 1 h at RT at manufacturer recommended concentrations. The membrane was then washed again and developed using the SuperSignal chemiluminescence substrate (ThermoFisher), and imaged using a ChemiDoc Imaging System (Bio-Rad).

### Cryo-transmission electron microscopy

Cryo-transmission electron microscopy (Cryo-TEM) images were acquired according to protocols published previously [28]. Briefly, the VLP specimen were prepared using thin-film plunge freezing in an FEI Vitrobot. The vitrified specimen was mounted onto a Gatan 626.DH cryo-holder and transferred into an FEI Tecnai F20 TEM. Cryo-TEM images were obtained using low-dose mode.

### Real-time PCR

The real-time PCR (RT-PCR) protocol was adapted from previous work [29]. To this end, the VLP sample was mixed at 1:1 ratio with 2× RNA lysis buffer containing 2% Triton X-100, 50 mM KCl, 100 mM Tris–HCl (pH 7.4), 40% glycerol, and 0.4 U/μl of Superase·In RNase inhibitor (ThermoFisher). After 10 min incubation at RT, the lysed sample was diluted 1:5000 in nuclease-free water, resulting in 1:10 000 final dilution. Quantitative RT-PCR was performed using the SuperScript™ III Platinum™ SYBR™ Green One-Step qRT-PCR Kit (ThermoFisher). Here, a 50-μl reaction mixture was prepared in 0.2-ml PCR tube containing 10 μl diluted VLP lysate, 1 μl SuperScript® III RT/Platinum® Taq Mix (includes RNaseOUT™), 25 μl 2× SYBR® Green Reaction Mix, 1 μl forward primer (5′-CCAGGAGTCAAATGGAAATTGAT-3′, 0.2 μM final concentration), 1 μl reverse primer (5′-CGATATGTTCGAAGGCATAGCC-3′, 0.2 μM final concentration), and 12 μl nuclease-free water. PCR was performed using a C1000 Touch Thermal Cycler (Bio-Rad) for 93-nucleotide product amplification. The cycling program was as follows: hold 50°C for 3 min; hold 95°C for 5 min; and 40 cycles of 95°C for 15 s and 60°C for 30 s. The CFX maestro software (Bio-Rad) was utilized to monitor SYBR Green signal. As relative mRNA expression level was compared among different VLP samples, no internal control was used. Three identical wells were tested in each run to account for technical replicates, and three independent runs were performed to account for biological replicates. The threshold was determined using auto mode in the software. Relative mRNA expression quantified the amount of RNA in 3P VLPs versus 4P VLPs based on measured ΔCt values: 2^ΔCt^ (ΔCt = Ct_4PVLPs_ − Ct_3PVLPs_).

### Droplet digital PCR

VLP lysate was prepared using 1:1 volumetric ratio with 2× RNA lysis buffer as described in the previous section. The lysed sample was then diluted to 1:1000 000 (final dilution) in nuclease-free water. The primer/probe mix directed against the viral PS9 sequence, containing forward primer: 5′-AGACAGTGGTTGCCTACG-3′, reverse primer: 5′-CAGTTGCACAATCACCAATCA-3′, and probe: 5′-AGATCTGAATCGACAAGCAGCGTACC-3′ produced by IDT (Coralville, IA) was resuspended in nuclease-free water at a 20× concentration. The One-Step RT-ddPCR Advanced Kit for Probes (Bio-Rad) was used to prepare a 20-μl reaction in a high-profile 96-well PCR plate (Bio-Rad). The reaction mixture contained 5 μl diluted VLP sample, 5 μl supermix, 2 μl reverse transcriptase, 1 μl 300 mM DTT (dithiothreitol), 1 μl 20× primer/probe mix (final 1× concentration at 500 nM primer, 250 nM probe), and 6 μl nuclease-free water. The plate was placed into the Automated Droplet Generator in a QX200 AutoDG Droplet Digital PCR System. Up to ∼20 000 droplets in each reaction were created using the default settings. Subsequently, the plate was foil-sealed and placed into a C1000 Touch Thermal Cycler (Bio-Rad) for reverse-transcription and amplification. The cycling program was as follows: 60 min at 50°C for complementary DNA synthesis; 10 min at 95°C for pre-denaturation; 40 cycles of 30 s at 95°C for denaturation; 60 s at 60°C for annealing/extension; and 10 min at the end at 98°C for enzyme deactivation. Droplets were then read using the QX200 Droplet Reader and the data were analyzed using the Bio-Rad QuantaSoft software v1.7. Droplets containing the viral RNA contribute to the positive population whereas those devoid of the viral RNA belong to the negative population. mRNA copy number per unit volume was calculated by the software, using default settings.

### SARS2 N protein ELISA assay

The VLP sample was lysed over 10 min at RT using 1:1 volume ratio with 2× lysis buffer containing 2% Triton X-100, 50 mM KCl, 100 mM Tris–HCl (pH 7.4), 40% glycerol, and 2× Halt protease inhibitor (ThermoFisher). The lysate was further diluted in nuclease-free water to obtain 1:1000 000 final VLP dilution. The LEGEND MAX™ SARS-CoV-2 Nucleocapsid Protein ELISA Kit (BioLegend) was used to quantify the N protein equivalent of the VLP samples, using calibration standard and instructions provided by the manufacturer. Absorbance at 450 nm was measured and VLP titer was calculated based on the standard curve.

### Dynamic light scattering

VLP samples were diluted 1:100 in PBS buffer. A total of 200 μl of this diluted sample was added into disposable plastic cuvettes and placed in a Zetasizer Ultra instrument (Malvern Panalytical, United Kingdom). In the ZS Xplorer software 3.0, materials was set to “liposomes”, dispersant was “water”, data processing was “general purpose”, and angle of detection was set to “back scatter”. All other parameters remained default setting. Particle diameter (in nm) was estimated by the software based on the measured scattering intensity (%).

### CRISPR–Cas9 VLP editing assay

In some studies, gene editing was performed on 293T-EGFP, 293T-hACE2, and 293T-hACE2-EGFP cells. sgRNAs against the target gene were designed using the CRISPRscan software [30], and cloned into pKLV-U6gRNA(BbsI)-PGKpuro2ABFP. These include 5′-GGCGAGGGCGATGCCACCTA-3′, 5′-GAGCTGGACGGCGACGTAAA-3′, and 5′-GAGAGTGATCCCGGCGGCGG-3′ against *EGFP*; 5′-TGGTATAGACTGCAGCCATC-3′ and 5′-TTCTGTGATACACACGGCTG-3′ against *SLC35A1*; and 5′-TGTCATTTCAGAATAATGCT-3′ and 5′-CACTTGCCCAAATGTATCCA-3′ against *hACE2*. All sgRNAs were simultaneously used on the target cells by either co-transfection with multiple plasmids or applying pooled lentivirus when stable cells were established. To this end, cells were transfected with pooled plasmids when targeting EGFP or hACE2 24–48 h prior to addition of Cas9 VLPs. 293T cells stably expressing sgRNA against *SLC35A1* were used when targeting endogenous gene. To introduce Cas9, 3P VLPs were prepared carrying either Cas9-P2A-dTo-PS9 (dTo:dTomato) or Cas9-P2A-dTo-T20, with SARS2 S or VSV-G viral glycoprotein as 100× concentrates. These VLPs were introduced into target cells in 1.5-ml microcentrifuge tubes by incubating 80 000 cells in 8 μl volume with varying amounts of VLPs in the presence of 8 μg/ml polybrene for 25 min at RT. Transduced cells were then transferred into six-well plates with the addition of 5 ml fresh DMEM media per well. Cells were cultured for 6 days with fresh media being added every 48 h. On day 6, gene editing efficiency was quantified using flow cytometer. Additionally, in studies that monitored hACE2 editing, the cellular genomic DNA was purified from individual treatments using the PureLink genomic DNA isolation kit (ThermoFisher). The region surrounding the hACE2 editing target site was PCR amplified using forward primer: 5′-TCAAGCAATGCCATTCCAACTTC-3′ and reverse primer: 5′-CTGAGAGCACTGAAGACCCAT-3′. Standard Nextera Index primers were then added using a second overlapping PCR and the product was subjected to 150-bp paired-end sequencing on a MiSeq instrument (Illumina, San Diego, CA). Sequencing results were demultiplexed and editing efficiency was analyzed using a custom python script that quantified indels at the edit site. Editing percentage quantifies the number of reads with indels/total number of reads.

### Animal study

All animal experiments were approved by the Veterans Administration of Western New York Healthcare System’s Institutional Animal Care and Use Committee (Buffalo, NY). CD-1 mice (weight = 20∼21 g, equal number of both sexes) were purchased from Charles River Laboratory. 3P VSV-G Luc-T20 and 3P mouse-adapted SARS2 (maSARS2) Luc-T20 VLPs were produced after 300-fold concentration. In studies with maSARS2, VLP amount was further adjusted based on N protein equivalent values. Mice were anesthetized in chamber using 3.0% isoflurane in 100% O_2_ at 1.5 l/min until unresponsive. Equal numbers of mice were left untreated (negative control), treated with VLPs via intranasal (i.n.) route or subjected to VLP instillation via oropharyngeal aspiration (o.p.a.). In the case of i.n., mice were grasped behind the neck and held vertically with nose up, and a total of 50 μl VLP solution was alternatively applied on both nares as the animal inhaled. In the case of o.p.a., mice were hung by their front teeth with a suture on a 60° incline board. The tongue was grasped with gauze sponge and pulled straight out. With the chest being squeezed, a first 50 μl VLP dose was deposited into the back of oropharynx and then the chest was released. Following the recovery of regular breathing, additional 50 μl VLP was similarly instilled. Mice were sacrificed at 24 h. The left lung, right lung, and trachea tissue of each mouse were obtained and put in tared 1.5-ml “Navy bullet” tubes (Next Advance, Troy, NY) separately. Enough ice-cold tissue lysis buffer (Gold Biotechnology) with Halt protease inhibitor cocktail (ThermoFisher) was added to the individual tube to yield a “tissue + fluid” total weight of 900 mg. Subsequently, the “Navy bullet” tubes were put in a Bullet Blender Storm 24 (Next Advance) in cold room and run at level-8 for 5 min twice. Homogenous supernatant was collected after spinning samples at 20 000 × *g* for 30 min at 4°C. Samples were stored at −80°C, prior to performing the luciferase assay as described previously, and measuring total protein concentration using the BCA Protein Assay kit (ThermoFisher). Luciferase signals reported in this manuscript were normalized based on BCA-based protein concentration measurements. These measurements did not vary by >20% among the different samples.

### Statistics

All data are presented as mean ± standard deviation (STD) for multiple biological replicates. Dual comparisons were performed using the Student’s two-tailed *t*-test. Multiple comparisons were performed using ANOVA followed by the Tukey post-test. *P-*value<.05 was considered to be statistically significant. Number of repeats is presented using discrete points in individual plots. Western blots are representative of multiple repeats. All samples in VLP infectivity assays were paired in order to account for biological variability.

## Results

### Establishing a “3P” SARS2 VLP system with improved viral entry properties

**Figure 1.**
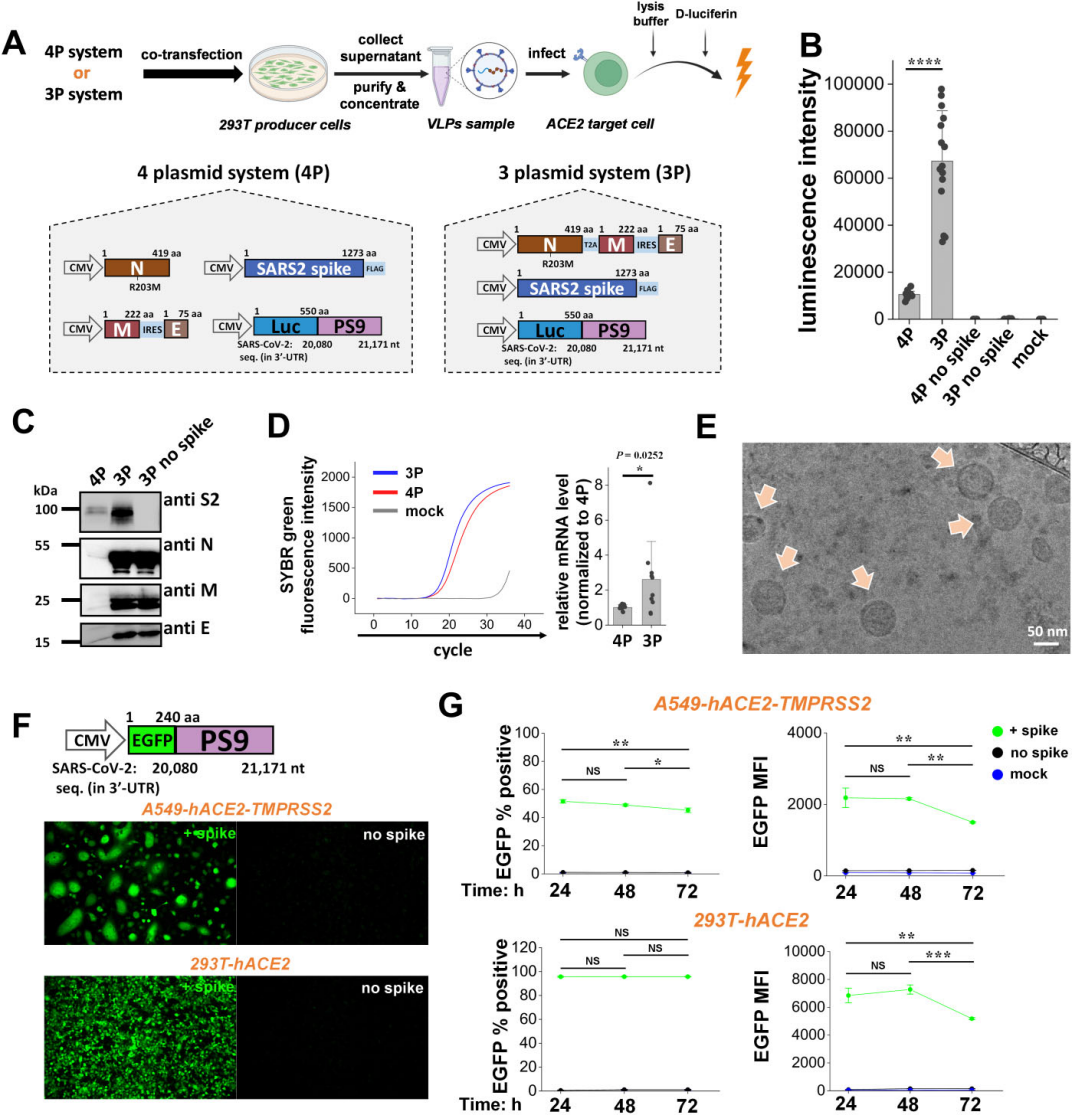
3P SARS2 VLPs are superior to 4P SARS2 VLPs. (**A**) 4P and 3P SARS2 Luc-PS9 VLPs were produced by co-transfection of either four or three plasmids into 293T producer cells. These plasmids encode for the viral structural proteins, along with a luciferase reporter coupled to PS9 packaging signal (Luc-PS9). (**B**) Luciferase assay showed ∼7-fold higher luminescence intensity in 293T-hACE2 cells upon using 3P versus 4P VLPs. (**C**) VLP concentrate (10 μl for M and E, 2 μl for S2 and N) was loaded in each lane. Western blot analysis suggests incorporation of all SARS2 structural components in VLPs, with 3P SARS2 Luc-PS9 VLPs displaying more intense protein bands compared with 4P VLPs. (**D**) Higher Luc-PS9 transcript levels were observed using RT-PCR in the case of 3P SARS2 Luc-PS9 VLPs. (**E**) Cryo-TEM images of 3P SARS2 Luc-PS9 VLPs showed spherical ∼100-nm-sized VLPs with double-layered membrane structures. (**F**) 3P SARS2 EGFP-PS9 VLPs produced with EGFP reporter efficiently infected 293T-hACE2 and A549-hACE2-TMPRSS2 cells. Fluorescence images were acquired 24 h post-infection. (**G**) Flow cytometry VLP entry time-course showed peak fluorescence for 3P SARS2 EGFP-PS9 VLPs at 24 h followed by decrease at larger times. This was observed both for 293T-hACE2 and A549-hACE2-TMPRSS2 cells. Abbreviations: aa, amino acid; nt, nucleotide. Data are mean ± STD. *P < .05, **P < .01, ***P < .001, ****P < .0001, NS: not significant.

As the co-transfection of four plasmids into single cells could result in a heterogeneous VLP population due to cell-to-cell variability in exogenous DNA expression, we sought to reduce the number of plasmids required for such particle production. A number of combinations were tested, with the final product containing the N protein linked via a T2A self-cleaving peptide upstream of the M-IRES-E gene cassette (IRES sequence from EMCV) (Fig. 1A). Co-transfection of this plasmid, along with plasmid encoding for SARS2 S and luciferase reporter linked to the PS9 packaging sequence resulted in functional 3P SARS2 Luc-PS9 VLPs capable of expressing reporter mRNA in recipient cells. As the number of plasmids used for VLP generation was reduced from four to three, this new system is called the “3P system”.

Spike plasmid transfection amounts were varied in 150-mm petri dishes to optimize functional VLP production (Supplementary Fig. S1A and B). Optimal 4P and 3P VLPs were formed upon using 1 μg spike plasmid along with 50 μg of plasmid encoding for other viral components. Thus, the stoichiometry of viral proteins in host cells dictated the efficacy of the produced VLPs. 3P VLPs not only resulted in a simpler VLP production workflow, but also displayed ∼7-fold higher luminescence intensity using 293T-hACE2 target cells compared with 4P VLPs (Fig. 1B). VLP entry was strictly spike dependent, as VLPs without spike did not induce luminescence signal in target cells. Western blot analysis showed the presence of all structural proteins for both the 4P and 3P VLPs: S2 (∼95 kDa), N (∼46 kDa), M (∼25 kDa), and E (∼10 kDa) proteins (Fig. 1C). 3P VLPs displayed more intense bands suggesting more uniform expression of structural proteins in producer cells. Real-time quantitative PCR analysis of VLP lysates suggests ∼3-fold higher expression of the PS9 transcript in the 3P VLPs, compared with 4P VLPs (Fig. 1D). Cryo-TEM showed that the 3P VLPs were uniform, 80–100-nm sized with prominent double layered membranes (Fig. 1E). Dynamic light scattering (DLS) showed that 3P SARS2 Luc-PS9 VLPs that carried the S protein were slightly larger (∼146 nm) compared with VLPs lacking S (∼125 nm) (Supplementary Fig. S1C).

EGFP-PS9 was introduced in place of Luc-PS9 to quantify infection in single cells using microscopy and flow cytometry. In microscopy studies, 3P SARS2 EGFP-PS9 VLPs infected considerable numbers of A549-hACE2-TMPRSS2 and 293T-hACE2 cells, both in a spike-dependent manner (Fig. 1F). Using flow cytometry for quantitation, we noted that ∼52% of A549-hACE2-TMPRSS2 and ∼96% of 293T-hACE2 cells were EGFP-positive 24 h post-infection (Fig. 1G). Fluorescence signal persisted up to 72 h, with 46% of A549-hACE2-TMPRSS2 cells, and 94% of 293T-hACE2 cells remaining EGFP-positive. However, a decrease in the measured signal intensity was observed at greater times in the cytometry histogram (Fig. 1G and Supplementary Fig. S1D), likely due to the degradation of EGFP mRNA and EGFP protein in recipient cells along with cell division which splits the signal among daughter cells. Overall, 3P VLPs were developed that both simplified SARS2 VLP usage and improved efficacy compared with previously used 4P VLPs.

### Tuning SARS2 VLP tropism by switching viral glycoprotein

**Figure 2.**
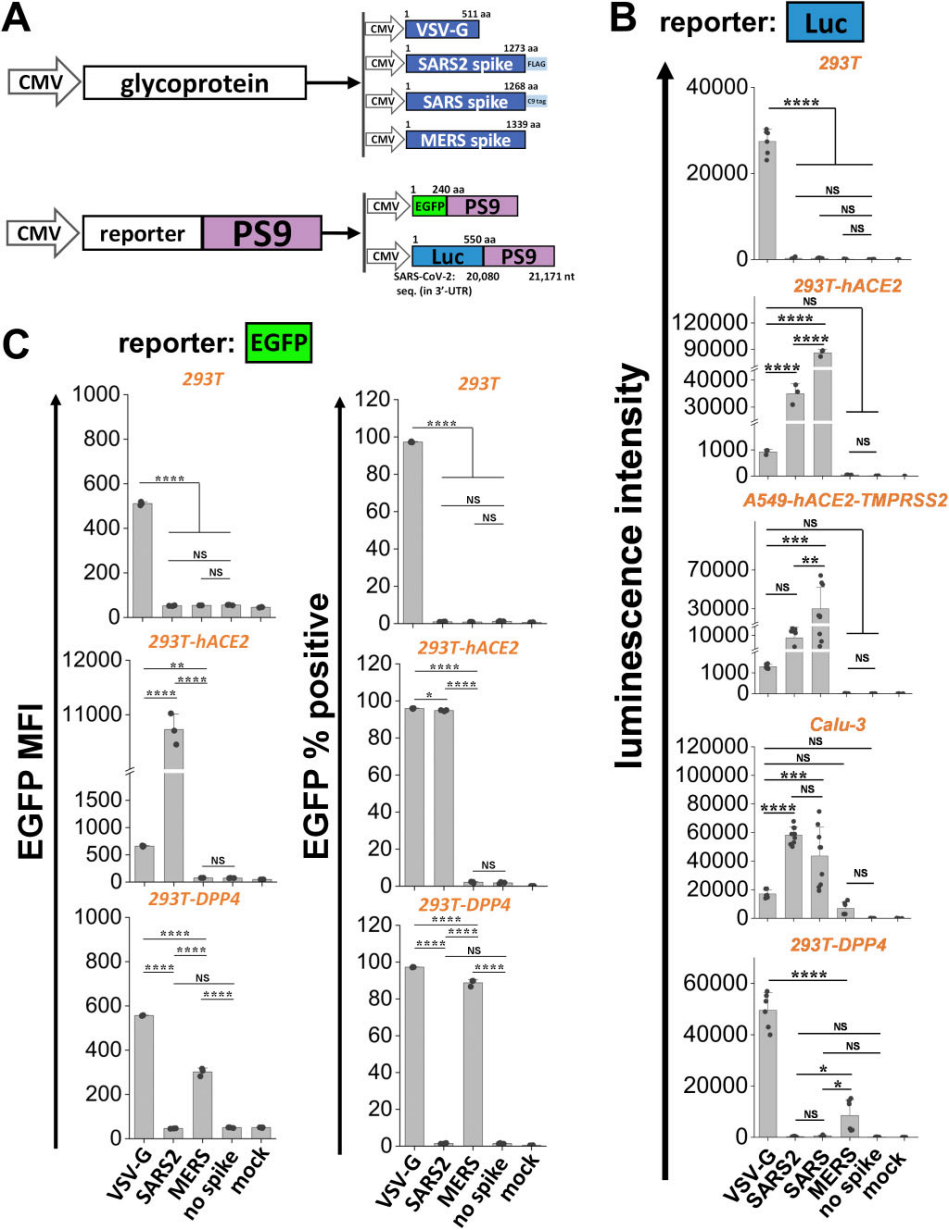
Tuning VLP viral tropism by altering viral glycoprotein. (**A**) Different types of VLPs were produced using the 3P system by varying the viral glycoprotein (VSV-G, SARS2 spike, SARS spike, or MERS spike) and reporter genes (luciferase or EGFP). (**B**) One microgram of the viral glycoprotein was used to create various 3P Luc-PS9 VLPs and these were used to infect five cell types: WT 293T (293T), 293T-hACE2, A549-hACE2-TMPRSS2, Calu-3, and 293T-DPP4. SARS2 and SARS spike VLPs displayed similar tropism and entered only hACE2-expressing cells (293T-hACE2, A549-hACE2-TMPRSS2, and Calu-3), with SARS exhibiting higher luminescence intensity compared with SARS2. MERS VLPs infected Calu-3 cell at low level and efficiently entered 293T-DPP4 cells. VSV-G VLPs entered all cell types. (**C**) 3P EGFP-PS9 VLPs were produced with 4 μg VSV-G, 1 μg SARS2 spike, or 1 μg MERS spike plasmid. VSV-G VLPs entered all cell types. SARS2 VLPs only entered ACE2 cells. MERS VLPs only entered DPP4 cells. Methods provide detailed steps for VLP production. Data are mean ± STD. *P < .05, **P < .01, ***P < .001, ****P < .0001, NS: not significant.

We determined if viral tropism could be tuned by altering the surface glycoprotein. In addition to SARS2 spike, this was tested using the VSV-G, and also betacoronavirus spike from the 2002 SARS and 2012 MERS virus (Fig. 2A). In this context, VSV-G binds low-density lipoprotein receptors and phosphatidylserine that are ubiquitously present on many mammalian cell types [31], and MERS spike recognizes DPP4 as host cell receptor [32]. To enable studies of MERS VLPs, isogenic HEK293T clones expressing DPP4 (293T-DPP4) were produced (Supplementary Fig. S2A) and Calu-3 was used as a lung epithelial cell model that is permissive to SARS, SARS2, and MERS spike [33, 34].

Initial studies were conducted using the 3P platform with Luc-PS9 reporter (Fig. 2B). Here, the 3P SARS2 Luc-PS9 VLPs and 3P SARS Luc-PS9 VLPs exhibited strict specificity for cells expressing hACE2, including 293T-hACE2, A549-hACE2-TMPRSS2, and Calu-3. The SARS VLPs displayed 1–3-fold higher infectivity for these cells compared with SARS2 VLPs. 3P MERS Luc-PS9 VLPs only infected 293T-DPP4 and Calu-3 cells. The 3P VSV-G Luc-PS9 VLPs infected all target cell types, albeit at lower levels compared with other VLPs. This was clear using both the standard sensitivity of the luminescence plate reader and upon increased detector sensitivity (Supplementary Fig. S2B). As expected, in the absence of spike, the VLPs failed to infect any of the cell types and the measured signal was similar to mock infection control. Titration studies were performed with VSV-G and MERS spike VLPs to determine optimal conditions for VLP production (Supplementary Fig. S2C and D). This revealed that the 3P VSV-G and MERS VLPs required greater plasmid mass of 4 and 2 μg, respectively, for optimal production as opposed to 3P SARS2 VLPs which only required 1 μg plasmid (Supplementary Fig. S1B).

Next, we evaluated the percentage of cells infected by these VLPs carrying the EGFP reporter (Fig. 2C). Here, 3P SARS2 EGFP-PS9 VLPs exclusively infected ACE2-expressing cells, while 3P MERS EGFP-PS9 VLPs only infected DPP4 cells. 3P VSV-G EGFP-PS9 VLPs infected all cell types at >90% efficiency (Fig. 2C), although the measured MFI was lower compared with 3P SARS2 EGFP-PS9 VLPs (Supplementary Fig. S2E). The differences observed in measured intensity across cell types for different VLPs, may be due to differences in the affinity of the VLP glycoprotein receptor for their ligand on host cells, and the expression levels of these ligands. This may cause variation in the number of EGFP mRNA copies and extent of measured fluorescence across infected cell types, even though most cells are infected. Overall, VLP tropism could be tuned to target specific recipient cell types. This feature may be exploited for the directed mRNA delivery to specific target cells.

### SARS2 VLP system could be streamlined into a 2P system

We determined if the number of plasmids could be further reduced from three to two, as this would further simplify downstream applications. Thus, all structural and reporter genes were expressed using a single vector, with a second vector being used for *trans*-complementation of the desired viral glycoprotein. To test this concept, initially, we replaced the NeoR/KanR resistance gene located downstream of the SV40 promoter in pcDNA3.1 CMV N-T2A-M-IRES-E with Luc-PS9 (Supplementary Fig. S3A). Titration experiments were performed by changing the ratio of this plasmid and the SARS2 spike plasmid over a wide range. The resulting VLPs infected 293T-hACE2 cells at levels above the no spike control, but the measured luminescence was low compared with the 3P SARS2 Luc-PS9 VLPs. We conjectured that this could be due to the low activity of the SV40 promoter or promoter interference within the single construct.

**Figure 3.**
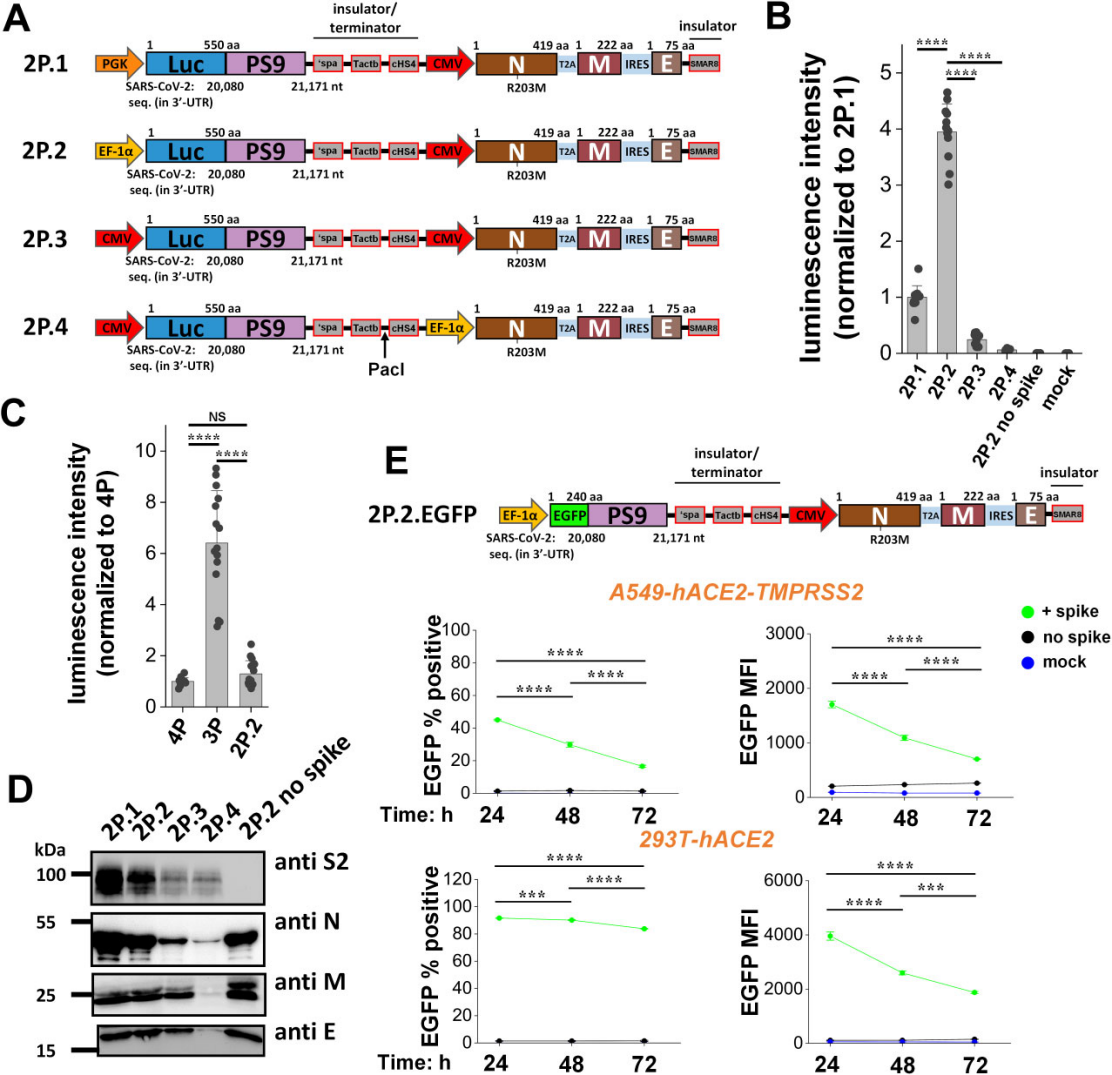
Streamlining VLP technology using a 2P system. (**A**) Four constructs were developed with two independent promoters driving expression of reporter gene and SARS2 structural proteins. The promoters were separated by insulator and terminator sequences to minimize promoter interference: synthetic polyA (spa); a G-rich sequence from β-actin (Tactb); chicken hypersensitive site 4 (cHS4); and a synthetic MAR sequence 8 (sMAR8) at the end of the E protein. (B, C) Each of these constructs was transfected into 293T cells along with spike plasmid to produce four different 2P SARS2 Luc-PS9 VLPs. 2P.2 VLPs displayed highest luminescence intensity (**B**). Its signal was comparable to 4P VLP but lower than 3P VLP (**C**). (**D**) Western blots of SARS2 structural proteins showed different patterns of protein expression for different 2P plasmids. 2P.2 SARS2 Luc-PS9 VLPs displayed more intense protein bands compared with 2P.3 and 2P.4, but this was lower than 2P.1. (**E**) 2P.2.EGFP VLPs were created by replacing the luciferase reporter with EGFP. VLP entry of 2P.2.EGFP SARS2 VLPs into A549-hACE2-TMPRSS2 and 293T-hACE2 cells was measured using flow cytometry. Data are mean ± STD. ***P < .001, ****P < .0001, NS: not significant.

To address the above limitations, we modified a previously developed LVDP vector, which can independently express two proteins driven by different promoters (Fig. 3A) [24]. This vector contains a series of insulator and terminator elements to minimize interference between the two promoters. Four different constructs (2P.1–2P.4) were developed, with PGK, EF-1α, or CMV promoter driving either the SARS2 structural proteins N, M, and E, or the Luc-PS9 reporter. All 2P SARS2 Luc-PS9 VLPs were produced and VLP entry was evaluated using 293T-hACE2 recipient cells (Fig. 3B). Here, the 2P.2 SARS2 Luc-PS9 VLPs showed high reporter signal comparable to 4P SARS2 Luc-PS9 VLPs, but this was lower than the 3P VLPs (Fig. 3C). VLP entry for all 2P VLPs was strictly spike dependent (Supplementary Fig. S3B). To rule out the possibility that promoter interference still exists in this system, we used the restriction enzyme *PacI* to linearize the vector between the two gene cassettes. Enzymatic digestion was confirmed using agarose gel electrophoresis of 2P vectors (Supplementary Fig. S3C). However, this treatment did not increase VLP entry function, as VLPs formed using the cut plasmids displayed ∼50% reduced signal in 293T-hACE2 cells compared with that of the uncut plasmids (Supplementary Fig. S3C). Upon analyzing SARS2 structural proteins using western blots, 2P.2 VLPs demonstrated stronger band intensities compared with 2P.3 VLPs and 2P.4 VLPs, but the measured signal was lower compared with the less infectious 2P.1 VLPs to some extent (Fig. 3D). The 2P.2 VLPs were sized similarly to the 3P VLPs based on DLS, with the spike bearing 2P.2 SARS2 Luc-PS9 VLPs being ∼146 nm in size compared with ∼125 nm for the same VLPs without spike (Supplementary Fig. S3D). Finally, in studies that titrated the spike plasmid mass, similar to the 3P VLPs, 1 μg spike plasmid mass was sufficient for maximal 2P.2 VLP infectivity (Supplementary Fig. S3E). These data suggest that the concentrations of different structural proteins and amount of Luc-PS9 mRNA may be regulated by swapping the promoters, and this is a key determinant of VLP function.

To study single-cell infection and perform time-course studies, the luciferase reporter in the 2P.2 plasmid was replaced by EGFP to create 2P.2 SARS2 EGFP-PS9 VLPs (Fig. 3E). Here, similar to studies with the 3P VLPs (Fig. 1G), reporter signal was maximum at 24 h, decreasing thereafter. 2P.2 SARS2 EGFP-PS9 VLPs infected 92% of the 293T-hACE2 and 45% of the A549-hACE2-TMPRSS2 cells at 24 h (Fig. 3E and Supplementary Fig. S3F). Overall, these experiments resulted in the 2P VLP system, with a simplified workflow and reduced number of reagents. These reagents reduce the workload for experimentalists as illustrated in an independent publication [35].

### SARS2 VLPs can package at least 5 kb mRNA and four transgenes

**Figure 4.**
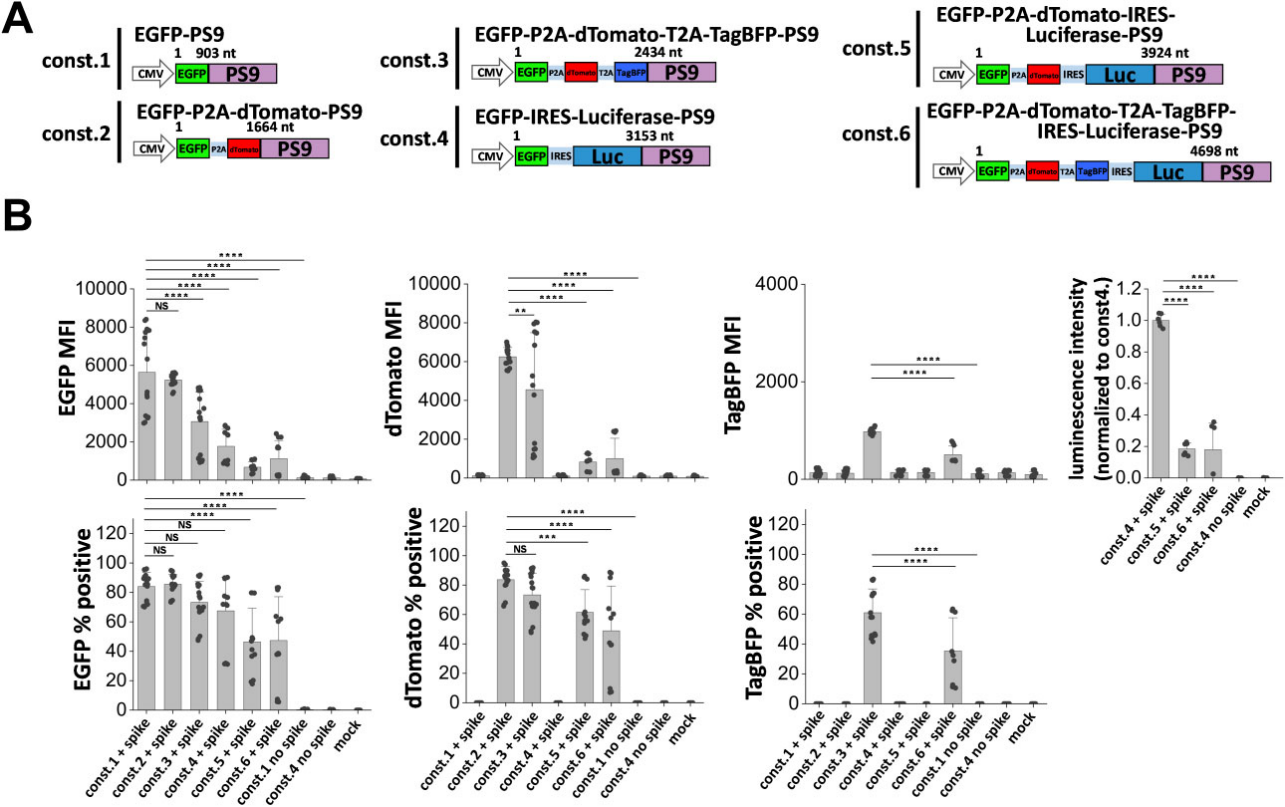
Delivery of four transgenes using 3P SARS2 VLPs. (**A**) Schematic of six PS9 constructs with successive addition of four different reporter genes: EGFP, dTomato, TagBFP, and/or luciferase. These constructs were employed to develop 3P VLPs for transgene delivery. (**B**) Fluorescence and luminescence signal for multiple biological replicates using different 3P constructs (same amount of VLP in each case). The relation between payload size and viral entry was measured based on reporter intensity (upper panels) and % fluorescent cells (lower panels). While percentage of infected cells decreased gradually upon increasing mRNA package size, the decrease in fluorescence intensity was more dramatic. Data are mean ± STD. **P < .01, ***P < .001, ****P < .0001, NS: not significant.

We investigated the packaging ability of the SARS2 VLPs in terms of the maximum mRNA size and number of gene payloads that can be delivered into target cells. To this end, a panel of six constructs were created with four reporter genes, EGFP, dTomato, TagBFP, and luciferase (Fig. 4A). These reporters were linked to a single CMV promoter either via T2A or P2A self-cleaving peptides, or IRES sequence. Thus, 3P SARS2 VLPs with payloads ranging 900–4700 nt complexed with the PS9 packaging sequence were evaluated for viral entry into 293T-hACE2 cells. Flow cytometry measured fluorescence reporter signal and a plate reader was used for luminescence measurement (Fig. 4B). All viral entry was spike dependent. While ∼85% of the cells carried the first EGFP reporter in the smallest const.1, this was reduced to 50% upon using the largest const.6 (Fig. 4B and Supplementary Fig. S4A). EGFP MFI also decreased with increasing payload size. Similar observations were made upon following other reporters including dTomato, TagBFP, and luciferase.

Several additional attempts were made to vary mRNA packaging efficiency while keeping spike plasmid constant, in order to enhance transduction into 293T-hACE2 target cells. In one aspect, we varied the concentration of the structural protein encoding plasmid and const. 6 reporter plasmid. This at most resulted in ∼25% signal improvement upon increasing reporter plasmid amount from 25 to 40 μg (Supplementary Fig. S5A). In a second case, we inserted a second PS9 sequence either at the 3′-end of the mRNA payload to generate the 3P SARS2 EGFP-PS9-PS9 VLP (Supplementary Fig. S5B), or at the 5′-end while varying the open reading frame to account for premature translation initiation. While the former manipulation resulted in percentage transduction comparable to the original 3P SARS2 EGFP-PS9 VLP, reporter signal intensity was reduced by ∼80% (Supplementary Fig. S5C). Surprisingly, the latter modification resulted in complete absence of reporter signal in recipient 293T-hACE2 cells. Overall, SARS2 VLPs were generated to carry payloads of ∼4.7-kb size, with a single promoter driving up to four transgenes.

### Changing payload size does not affect VLP synthesis

**Figure 5.**
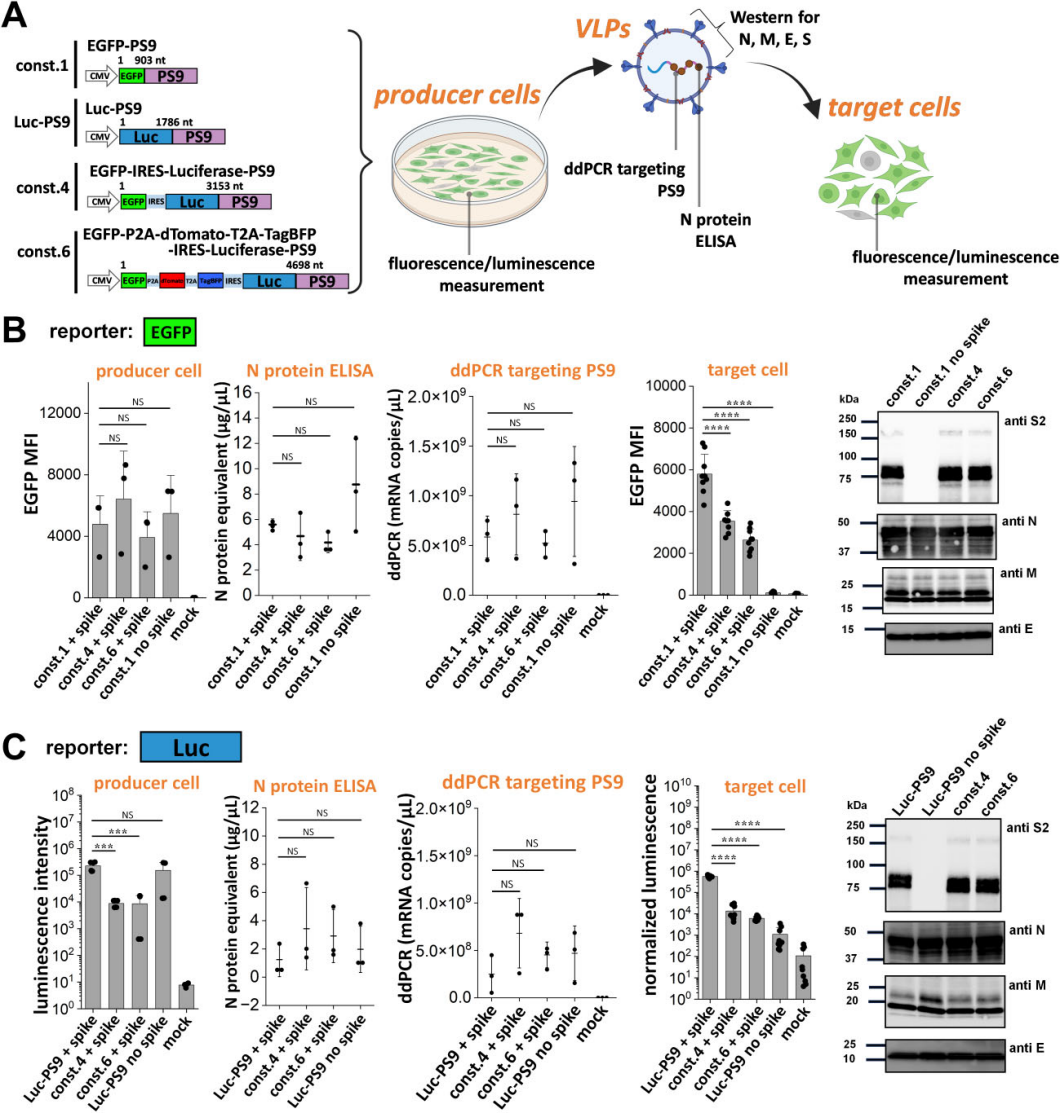
Mechanisms regulating VLP function and efficacy. (**A**) VLPs with either EGFP reporter (downstream of CMV promoter) or luciferase reporter (downstream of CMV or IRES) were produced using the 3P system. Various parameters were measured as illustrated. (**B**, **C**) The individual panels in these plots from left to right present: (i) reporter signal intensity in producer cells; (ii) N protein ELISA levels for individual VLP preparations; (iii) ddPCR quantitation of PS9 mRNA levels in VLPs; (iv) reporter signal in target cells following VLP infection; and (v) western blot of VLP structural proteins. EGFP fluorescence data are presented in panel (B) and luciferase reporter data in panel (C). The data showed that VLPs with similar structural compositions and mRNA copy numbers were produced for all payloads. However, increasing payload size progressively decreased reporter signal in target cells. Data are mean ± STD. ***P < .001, ****P < .0001, NS: not significant.

To investigate the mechanism(s) contributing to the decreasing reporter signal with increasing payload size, we systematically compared the impact of four different PS9 coupled payloads on reporter expression in producer cells, the VLP composition including the amount of packaged RNA using droplet digital PCR (ddPCR), and corresponding reporter expression in target cells (Fig. 5A and Supplementary Fig. S6A). For such work, N protein amounts in each VLP preparation was also monitored using enzyme-linked immunosorbent assay (ELISA) as this allows quantification of VLP amounts in “N protein equivalent” units (Supplementary Fig. S6B). The four VLPs include const.1, 3P SARS2 Luc-PS9 VLPs, const.4, and const.6, ranging in size from 903 to 4698 nt. The analysis revealed several notable findings: (i) The mechanism regulating EGFP expression in target cells is different from luciferase. This is because EGFP is always the first reporter with its expression being controlled by CMV in all constructs. Luciferase expression, on the other hand, was more highly expressed in Luc-PS9 due to the CMV promoter and it was lower for constructs (#4, #6) containing the ribosome entry IRES (Fig. 5B and C, 1st column). These observations are consistent with previous work showing low efficiency of IRES-dependent translation compared with the first Cap-dependent promoter activity [36]. (ii) Virus payload size did not impact either VLP physical particle numbers (measured using N protein ELISA, 2nd column) or amount of mRNA encapsulated in virus (measured using ddPCR, 3rd column). (iii) Western blots showed that RNA payload did not affect VLP structural proteins (5th column). (iv) Whereas EGFP signal did not vary with construct size in producer cells, it was reduced in the target cells. Thus, the mRNA may undergo 5′-truncation or other impairments during VLP assembly upon increasing payload size. Overall, while the VLPs are well formed independent of construct size, the number of functional mRNA is reduced upon increasing construct size.

### SARS2 VLPs can be used to deliver functional Cas9 mRNA for genome editing

We determined if SARS2 VLPs could be used for gene editing. Thus, we created 3P SARS2 VLPs that carried either Cas9-P2A-dTomato-PS9 mRNA or Cas9-P2A-dTomato-T20 mRNA (Supplementary Fig. S7A). Titration studies measured VLP entry into 293T-hACE2 cells for both constructs. While VLP dose-dependent entry was observed in both cases, T20 was a superior packaging signal compared with PS9 (Supplementary Fig. S7B). In luciferase-based reporter assays, also, 3P SARS2 Luc-T20 VLPs resulted in ∼2.8-fold higher entry signal in 293T-hACE2 cells compared with 3P SARS2 Luc-PS9 VLPs (Supplementary Fig. S7C). Thus, we used T20 as the packaging signal in downstream studies.

**Figure 6.**
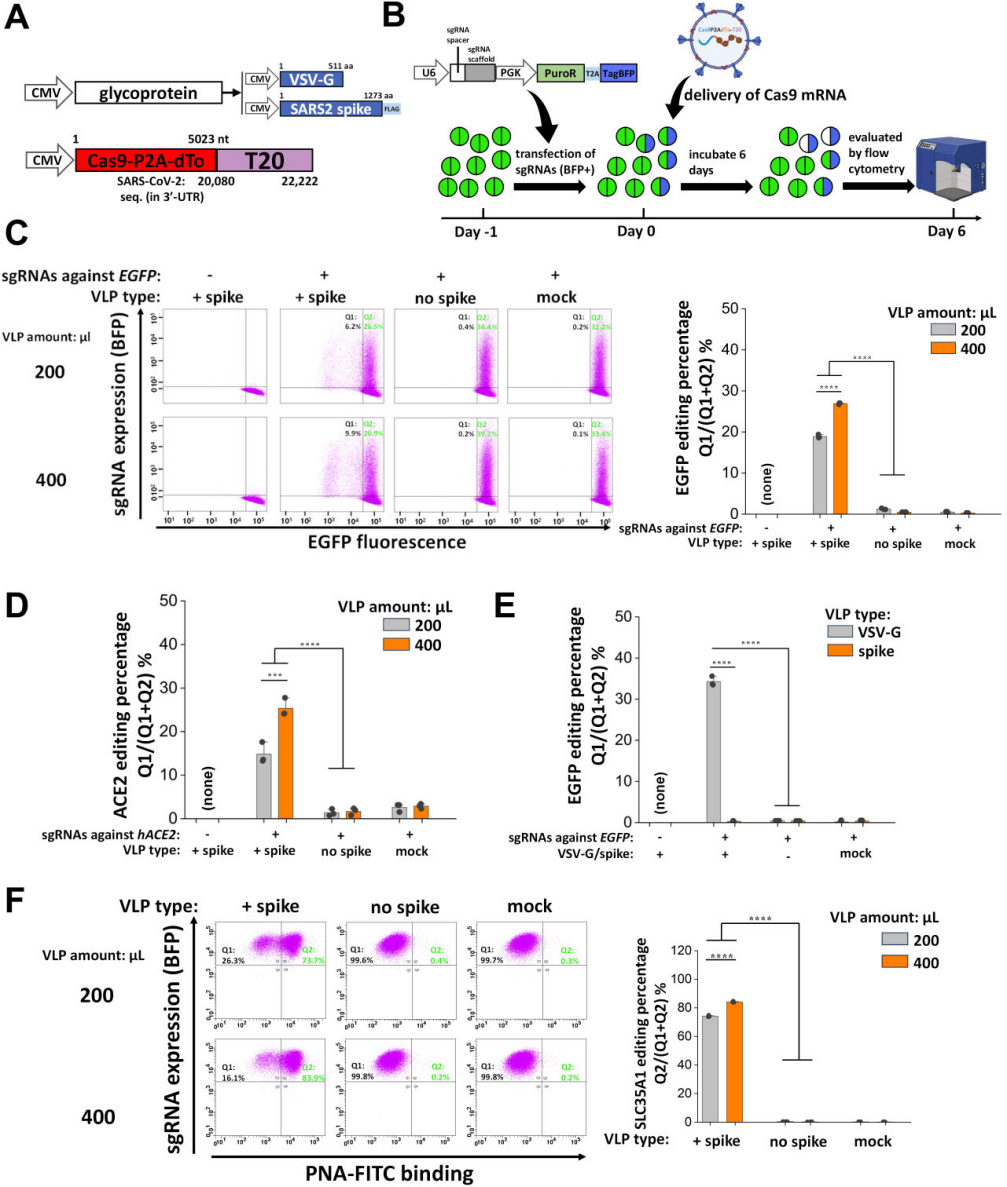
VLPs deliver functional Cas9 mRNA into target cells to achieve gene editing. (**A**) 3P Cas9-P2A-dTo-T20 VLPs (dTo: dTomato) were used for gene editing studies, with surface glycoprotein encoding for either VSV-G or SARS2 spike. (**B**) General workflow of gene editing study performed in panels (C)–(E). sgRNAs were transfected into cells on day −1 using plasmids carrying BFP reporter. VLP-carrying spCas9 mRNA was introduced into cells on day 0. Tropism of the VLP depends on surface glycoprotein. Gene editing efficiency was quantified on day 6. Editing efficiency quantified percentage of BFP(+) population that either turned EGFP(−) (panels C and E) or lost ACE2 expression based on anti-hACE2 binding (panel D). (**C**) sgRNAs targeting *EGFP* were introduced into 293T-hACE2-EGFP and spCas9 mRNA was delivered using 3P SARS2 Cas9-P2A-dTo-T20 VLPs. (**D**) sgRNAs against *hACE2* were introduced to knockout the receptor in 293T-hACE2 cells using 3P SARS2 Cas9-P2A-dTo-T20 VLPs. (**E**) sgRNAs targeting *EGFP* were introduced in 293T-EGFP cells, with genome editing being performed using 3P Cas9-P2A-dTo-T20 VLPs bearing either VSV-G or SARS2 spike. In all panels, the target gene (*EGFP* or *hACE2*) was knocked out in 20%–35% of cells expressing sgRNA. Higher VLP amount resulted in greater editing. (**F**) 293T-hACE2 stably expressed sgRNAs against *SLC35A1* were infected with 3P SARS2 Cas9-P2A-dTo-T20 VLPs (1.885 μg/μl N protein equivalent) or without SARS2 spike (1.385 μg/μl N protein equivalent). Gene editing efficiency was evaluated based on increase in fluorescent peanut agglutinin lectin (PNA) binding to cells. More than 70% gene editing was observed upon using 3P VLPs to edit endogenous genes. Volume of VLP used in each assay is specified in individual panels. Data are mean ± STD. ***P < .001, ****P < .0001, NS: not significant.

To determine if Cas9 mRNA delivered using the VLPs can mediate gene editing, two isogenic clones stably expressing high levels of EGFP were created using either 293T-hACE2 (293T-hACE2-EGFP; Supplementary Fig. S8A, left panel) or WT 293T cells (293T-EGFP;, Supplementary Fig. S8A, right panel). These cells were used for Cas9 editing assays using VLPs that contained Cas9-P2A-dTomato-T20 mRNA with either the SARS2 S or VSV-G glycoprotein (Fig. 6A). The ability of these VLPs to edit different cell types and genes was assessed (Fig. 6B). In one experiment, 293T-hACE2-EGFP cells were transfected with sgRNAs against EGFP gene (Fig. 6C). The transfected cells expressed blue fluorescence due to the presence of BFP reporter in the sgRNA vector [37]. The next day, the 3P SARS2 Cas9-P2A-dTomato-T20 VLPs were applied. Editing efficiency was quantified 6 days later by assessing EGFP levels in BFP-positive cells. Here, ∼20%–30% gene editing of BFP-positive cells was observed upon using VLPs bearing SARS2 spike, but not VLPs produced in the absence of spike. In other negative controls, cells lacking sgRNA and mock infected cells without VLPs failed to contain gene edits.

We examined if the above methodology can be extended to “self-inactivate” the hACE2 receptor (Fig. 6D and Supplementary Fig. S8B). Thus, sgRNAs against hACE2 was delivered using the same BFP reporter vector, this time, into nonfluorescent 293T-hACE2 cells. Here, also, 15%–25% knockout of cell surface ACE2 was observed at day 6 upon using 3P SARS2 Cas9-P2A-dTomato-T20 VLPs, but not upon using VLPs lacking SARS2 spike, cells lacking sgRNA against ACE2, or mock infected cells. To rule out any possible artifact, besides cytometry, gene editing was also confirmed using Illumina next-generation sequencing by performing amplicon sequencing of the editing sites on the hACE2 gene (Supplementary Fig. S8C). The results quantitatively agree with the cytometry results based on the percentage reads with gene edits, after accounting for the fraction of cells that are BFP-negative.

Gene knockouts could be targeted to specific cell types by tuning VLP tropism. To demonstrate this, two types of VLPs were created carrying the VSV-G (3P VSV-G Cas9-P2A-dTomato-T20 VLPs) and SARS2 S glycoprotein (3P SARS2 Cas9-P2A-dTomato-T20 VLPs). Both VLPs were applied to 293T-EGFP that lack the hACE2 receptor, but transiently expressed sgRNAs against EGFP using the above BFP reporter vector (Fig. 6E and Supplementary Fig. S8D). As anticipated, while the VSV-G VLPs could mediate ∼35% gene editing based on the measured decrease in EGFP fluorescent cells at day 6, editing was absent upon using SARS2 VLPs, in the absence of sgRNAs and in mock infection controls. Notably, while dTomato signal was observed in recipient cells at 24 h, this signal was absent at day 6, confirming that payload delivery using SARS2 VLPs was transient and nonintegrative (Supplementary Fig. S8E).

We determined if endogenous genes could be knocked out using this approach, in addition to reporter genes in above studies. To test this, we knocked out *SLC35A1* (CMP-sialic acid transporter) as absence of cell-surface sialic acid exposes galactose terminated glycans that can be readily detected using lectins like PNA [38] (Supplementary Fig. S9A). Thus, 293T-hACE2 cells stably expressing sgRNAs against *SLC35A1* were established, using co-expressed BFP as a selection marker (Supplementary Fig. S9B). Infection of 3P SASRS2 Cas9-P2A-dTomato-T20 VLPs into these “293T-hACE2-SLC35A1 sgRNA” cells resulted in >70% gene editing when using 1.885 μg/μl N protein equivalent VLPs, while this further increased to ∼85% upon doubling VLP volume (Fig. 6F). In comparison, transfection of Cas9-P2A-dTomato-T20 plasmid into these cells resulted in ∼65% gene editing (Supplementary Fig. S9C). In negative control no spike VLP (1.385 μg/μl N protein equivalent) failed to edit the cells. Overall, SARS2 VLPs can carry functional 5 kb Cas9 mRNA for gene editing in a target cell type-specific manner.

### SARS2 VLPs could be used for *in vivo* delivery into mouse lung

**Figure 7.**
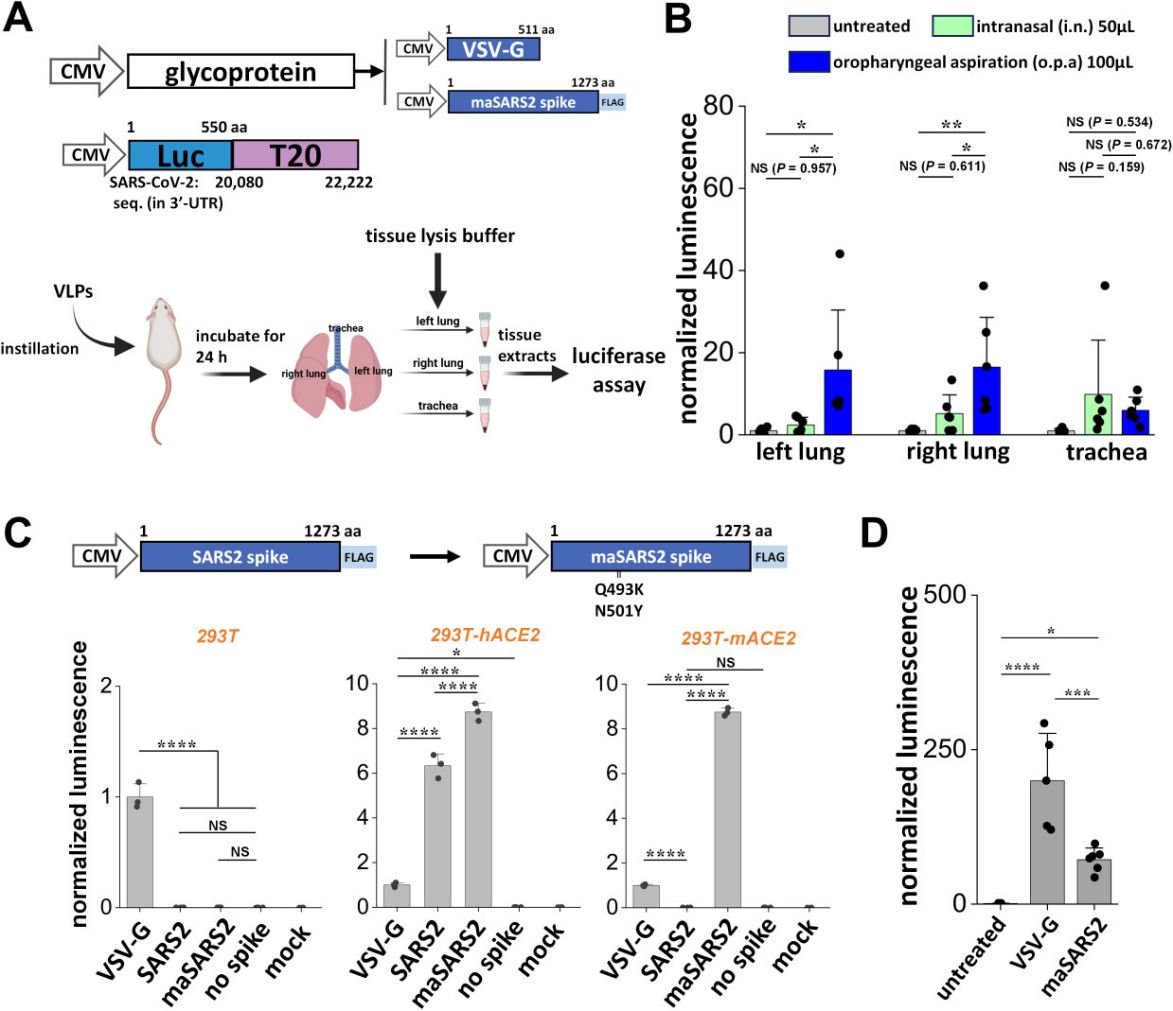
*In vivo* pulmonary gene delivery using VLPs. (**A**) VLPs bearing either VSV-G or maSARS2 were instilled into mice at time = 0. Twenty-four hours post-instillation, luciferase activity was measured in tissue extracts from left lung, right lung, or trachea. Protein concentration in lysate was used to normalize luminescence signal, with untreated/mock values being set to 1.0 for all *in vivo* studies. (**B**) VSV-G VLPs were instilled via either o.p.a. (100 μl) or i.n. (50 μl) routes. o.p.a. resulted in VLP administration to mouse lung. (**C**) Q493K and N501Y mutations were introduced into SARS2 spike to generate maSARS2 spike. 3P VLPs with VSV-G, SARS2, maSARS2, and no spike were produced with N protein equivalents of 1.859, 1.149, 1.334, and 2.024 μg/μl, respectively. A total of 50 μl of VLP was used to infect three target cell types: 293T, 293T-hACE2, and 293T-mACE2. VSV-G VLPs infected all three cell types, SARS2 spike only infected 293T-hACE2 (hACE2), whereas maSARS2 spike was permissive to both 293T-hACE2 and 293T-mACE2. VSV-G luminescence was set to 1.0 in this panel. (**D**) Hundred microliters of VSV-G VLPs or maSARS2 VLPs, both 0.820 μg/μl N protein equivalent, were instilled via o.p.a. into mice. Whole lung tissue was harvested. Mice without VLPs served as negative control. Both VSV-G and maSARS2 VLPs enabled luciferase signal in mouse lung with VSV-G being more efficient. Data are mean ± STD. *N* = 5–6 for each mouse treatment group. *P < .05, **P < .01, ***P < .001, ****P < .0001, NS: not significant.

As SARS2 is a pulmonary virus, we determined if these VLPs can overcome physiological barriers in the mouse lung to enable mRNA delivery (Fig. 7A). In one study, we produced 3P VSV-G Luc-T20 VLPs and instilled them into the mouse lung via either o.p.a. (100 μl) or i.n. (50 μl) routes. A third group without VLPs served as negative control. All mice were sacrificed at 24 h, lungs (left and right) and trachea were harvested, and luciferase activity was measured in tissue lysates. Here, o.p.a. allowed more specific VSV-G VLP delivery to the lungs (Fig. 7B).

Next, to enable spike-dependent VLP delivery, “maSARS2 spike” was developed by introducing Q493K/N501Y mutations into the natural spike as it does not bind mouse ACE2 [39] (Fig. 7C). 293T cells stably expressing mouse ACE2 receptor (293T-mACE2) were produced (Supplementary Fig. S10), and indeed these were permissive to 3P maSARS2 Luc-T20 VLPs (Fig. 6C). In contrast, 293T-hACE2 cells were infected by both 3P SARS2 VLPs and 3P maSARS2 VLPs. As anticipated, the VSV-G VLPs infected all cell types. Next, equal N protein equivalents of 3P Luc-T20 VLPs bearing either VSV-G or maSARS2 were instilled into mouse via o.p.a route (Fig. 7D). Following this, we noted higher delivery of luciferase reporter in mouse lung using VSV-G VLPs compared with maSARS2 spike VLPs, likely due to more ubiquitous expression of the lipoprotein receptor compared with mouse ACE2 [31]. Thus spike ACE2-dependent *in vivo* mRNA delivery was possible in lungs.

## Discussion

This manuscript advanced SARS2 VLP technology by exploring questions related to (i) the design of plasmids necessary for efficient production of these particles, (ii) methods to tune the tropism of the VLPs, (iii) optimizing the packaging capacity and characterizing the particle formation of the VLPs, (iv) gene editing applications, and (v) *in vivo* pulmonary delivery.

In one aspect, the study presents approaches to simplify the production of SARS2 VLPs, in order to produce more uniform particles. Decreasing the number of plasmids from four (4P system) to three (3P system) led to a less complex VLP synthesis process and at the same time markedly increased reporter signal by ∼7-fold. Further decreasing the number of plasmids to two (2P system) resulted in VLPs with reporter signal comparable to the 4P VLPs. As 2P VLP efficiency was lower than that of 3P VLP, and as the composition and reporter signal varied with the utilized plasmid promoter, we surmise that the concentrations of SARS2 structural proteins in producer cells impact VLP function. Despite the lower efficacy, the 2P system drastically simplified experimental workflows during biological investigations [35]. Consistent with previous work [9], spike concentration in a narrow range was necessary for optimal VLP production. Whereas only 1/50 of the total plasmid in the transfection mix encoded for SARS2 spike during the production of optimal SARS2 VLPs, this fraction was 1/25 for MERS VLPs and 1/10 when producing VSV-G pseudotyped VLPs.

VLP tropism could be tuned by swapping the SARS2 S protein with spike from the 2002 SARS virus, the 2012 MERS virus, and the VSV-G glycoprotein. This allowed selective VLP delivery into permissive target cells, relying solely on the presence of the host cell receptor on the cell surface. Indeed, similar efforts have been made for targeted delivery of lipid or polymeric nanoparticles, e.g. by conjugating a sialic acid-binding “GALA” peptide onto EGFP–mRNA polyplexes for engaging dendritic cells [40], or surface-decorated exosomes with E3 aptamer for siRNA (small interfering RNA) delivery to prostate cancer cells [41]. While success has been demonstrated *ex vivo*, clinical translation is complicated in part due to high technical demand needed to make such particles [17, 18, 42]. Additionally, a protein corona commonly coats nanoparticles in complex biological milieu resulting in either loss of nanoparticle targeting ability *in vivo* and/or redirection to the liver [43]. Nevertheless, efforts continue in this area to promote nonhepatic mRNA delivery either by noncovalent attachment of antibodies [44] or surface modifications to modify the protein corona [45]. In contrast to these synthetic methods, viral tropism has evolved with high efficiency in nature. In addition to targeting a given organ, these VLP particles can be designed to engage specific cell types. Exploiting this using the SARS2 VLP platform may enable novel strategies for targeted gene and protein delivery.

In studies that examined the packaging capacity of the SARS2 VLPs, we did not establish the maximum packaging capacity of the VLPs though increasing the size of the packaged mRNA decreased payload expression level. In this regard, increasing payload size from ∼0.9 to ∼5 kb decreased reporter signal by ∼50%–90%. Mechanistic investigations suggest that increasing payload size does not affect VLP synthesis or RNA incorporation into these particles but may affect protein production in target cells via mechanisms that require additional investigation. Nevertheless, packaging of functional *S. pyrogenes* Cas9 (∼5 kb) was possible. Among the packaging sequences, we consistently observed that T20 was superior to PS9. This observation is somewhat different to the findings reported by Syed *et al.* [9], perhaps due to the use of different plasmid constructs. In this regard, we note that recent studies by Terasaki *et al.* suggest that critical portions of the SARS2 packaging signal may reside outside T20 and PS9 [46]. These authors suggest the presence of a distinct 1.4-kb-long *cis-*acting RNA packaging sequence in the nonstructural protein 12- and nonstructural protein 13-coding regions [46]. Additional studies are needed to weight the relative importance of different proposed packaging sequences, and to test the possibility that the packaging signal of SARS2 is not continuous. Such design that accounts for multiple segments of the viral genome may synergically promote mRNA package size and viral assembly efficiency.

Despite the low Cas9-dTomato reporter signal intensity, SARS2 VLPs encapsulating *S. pyrogenes* Cas9 mRNA (∼5 kb insert) were remarkably proficient at performing genome editing. Reporter genes expressed in cells at variable levels could be knocked out by >30% in the infected cells, while this fraction increased to >70% when targeting the endogenous gene *SLC35A1* using efficient sgRNAs. The level of editing is comparable to the lentivirus-like bio-nanoparticle system reported by Lu *et al.* [47]. Varying the sgRNA specificity allowed editing of different gene targets and modification of VLP tropism enabled editing in different cell types. It is noteworthy that Cas9 mRNA delivered using VLPs is only transiently expressed, making it susceptible to degradation by cellular RNases. The short half-life of mRNA both reduces potential off-target editing and minimizes the chance of deleterious gene integration [48]. Unlike DNA payloads, the nuclear translocation and transcription is not needed with mRNA, making protein expression and drug delivery more straightforward [49].

mRNA reporters could be delivered to mouse lung using VLPs bearing both VSV-G and maSARS2 S glycoproteins. Thus, the particles can mediate pulmonary gene delivery, overcoming barriers including the lung cilia, mucus, and humoral immunity [50, 51]. A variety of additional approaches, like small molecules that reduce mucin glycoconjugates may also be applied in parallel to further enable clinical translation [52]. While the focus of this study is on pulmonary delivery due to the natural tropism of the virus, the specific target cell type may be varied by tuning VLP tropism. The levels of mucosal antibodies against spike is also low even in COVID-19-immunized individuals [51], making this an attractive vehicle for mRNA delivery. Additionally, while the luminescence measurements in this study were made using whole tissue lysates, it is likely that the measured signal would be amplified if these same measurements were made using lung epithelial cells dissociated from the larger organ. The demonstration that mRNA can be delivered to lungs, holds promise for nonintegrative gene therapy to the lung both in the context of rare pulmonary genetic disorders like cystic fibrosis and lymphangioleiomyomatosis and more common disorders like chronic obstructive pulmonary disease [53, 54].

A limitation of this work is that while Cas9 mRNA was delivered using VLPs, sgRNA was delivered separately using an independent plasmid vector or using lentivirus. This is similar to the approach of Yin *et al.* who use one vehicle to deliver Cas9 mRNA and a different one for sgRNA and homology-directed repair template [55]. We are currently exploring approaches reported in literature to overcome this barrier. First, sgRNA and Cas9 are being introduced into VLPs using two plasmids, both with 3′ PS9 or T20 packaging signals. Co-transfection of these constructs along with other 3P plasmids may enable co-packaging of both mRNA in a single VLP. This approach is supported by the work of Mali *et al.* who demonstrated that 3′-modification of tracrRNA (trans-activating CRISPR RNA) may not affect sgRNA function [56]. Second, Cas9 is being fused with viral structural proteins to co-package Cas9/sgRNA RNPs in the delivery vehicle. This is similar to the gag–Cas9 fusion approach used in lentivirus by Hamilton *et al.* [57], and gag–base editor fusion in murine leukemia virus by Banskota *et al.* [20]. Third, aptamer/aptamer-binding protein (ABP) pairing approaches are being employed to co-package VLP structural proteins that are fused with ABP complexed with sgRNA that are 3′-modified with the reciprocal aptamer sequences. This is inspired by the work of Knopp *et al.* [58] who use MS2 coat protein to bind 3′-modified sgRNA containing its ligand and Lyu *et al.* [59] who demonstrate similar function using *com*-modified sgRNA.

Overall, this paper presents advances in the SARS2 VLP system both for basic science virology studies and mRNA delivery applications. It demonstrates the packaging of multiple genes in a single vector, the ability to perform genome editing, and *in vivo* gene delivery to the lungs. By exploiting the natural tropism and size features of the SARS2 virion, these VLPs may enable new applications that are not possible using conventional targeted drug delivery approaches.

## Supplementary Material

gkaf133_Supplemental_File

## Data Availability

Plasmids are available from Addgene. NGS data are deposited as part of NCBI BioProject: PRJNA1140657. All other data are available in the main text or in online supplement.
