# Supplementary material for: Tuning the tropism and infectivity of SARS-CoV-2 virus-like particles for mRNA delivery
Source: Nucleic Acids Res. 2025 Mar 4;53(5):gkaf133. doi: 10.1093/nar/gkaf133 (PMC11879429; doi:10.1093/nar/gkaf133)
Supplement: gkaf133_Supplemental_File [file gkaf133_supplemental_file.pdf]

Supplementary Materials

Yang et al.

Contents:

Supplementary Figures S1-S10

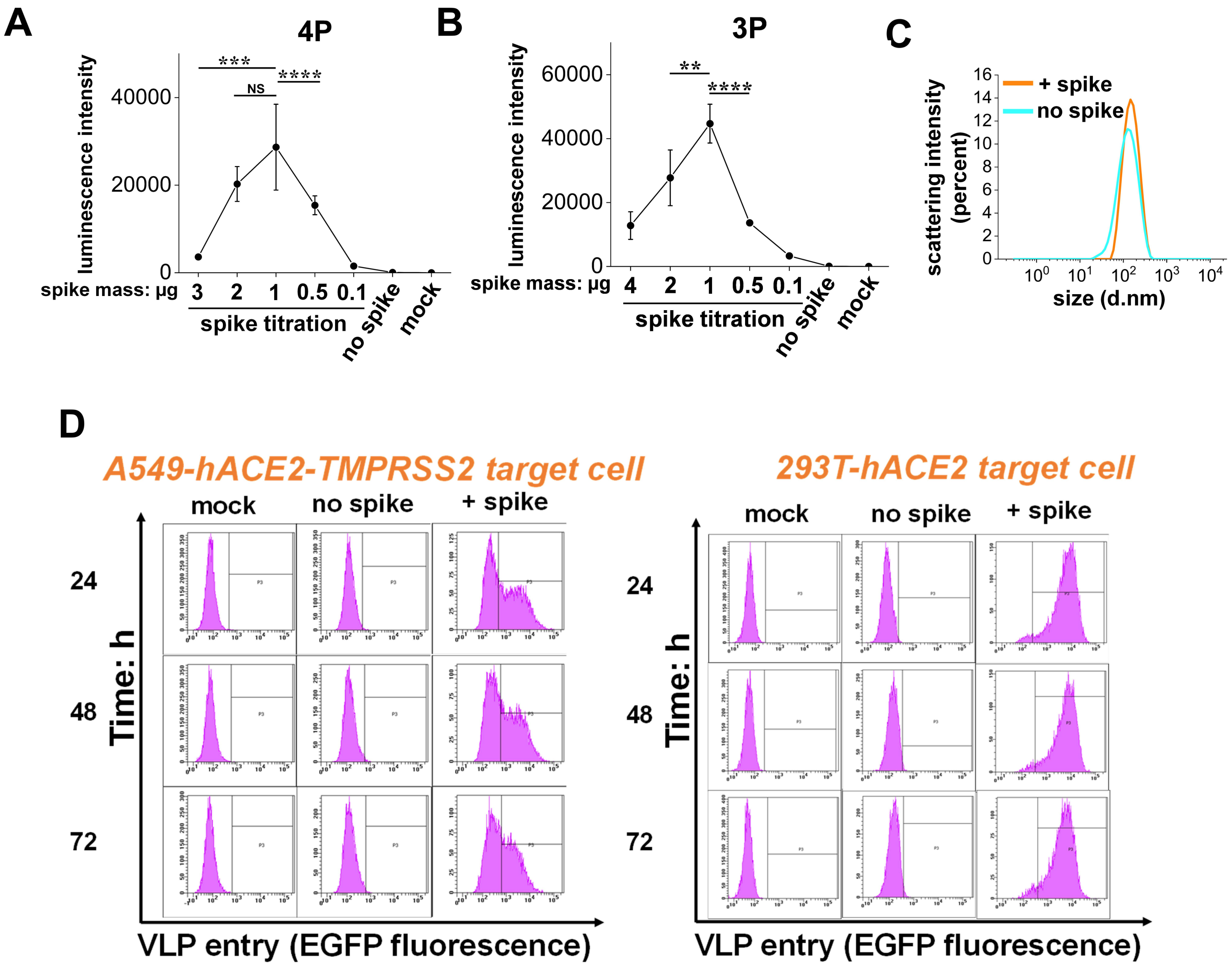

Figure S1. VLP system requires a specific spike plasmid mass for efficient production (Related to Figure 1). A-B. SARS2 Luc VLPs were produced for 4P VLPs (**panel A**) and 3P VLPs (**panel B**) in 150mm petri dishes, keeping the total plasmid mass constant at 50 µg/plate in all cases, while varying the proportion of spike plasmid from 0-4 µg. VLP entry properties was evaluated by measuring luminescence in recipient 293T-hACE2 cells. Highest entry peaked upon using 1 µg spike plasmid for both systems. **C.** Dynamic light scattering (DLS) quantified particle size distribution of 3P SARS2 Luc-PS9 VLPs. VLPs with spike sized at ~146 nm, slightly higher than that of the VLPs without spike (~125 nm). **D.** Representative flow cytometry histograms for data presented in **Figure 1G** (main Figure). Appreciable EGFP expression was observed in a vast majority of the cells, in these studies that measured 3P SARS2 EGFP-PS9 VLP entry into either 293T-hACE2 or A549-hACE2-TMPRSS2 cells. Data are Mean ± STD. $^{**}P < 0.01$, $^{***}P < 0.001$, $^{****}P < 0.0001$, NS: not significant.

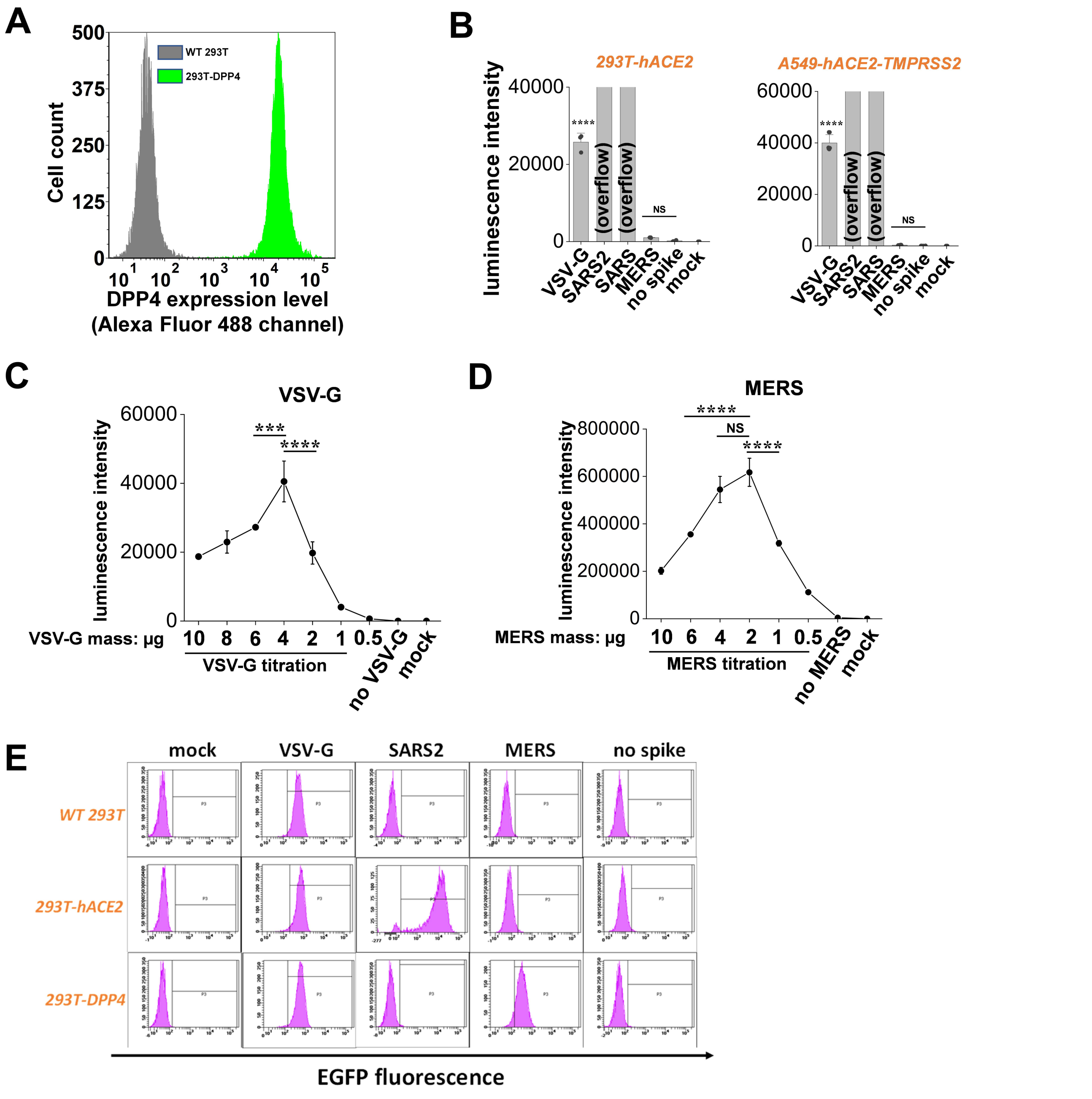

Figure S2. Tuning viral tropism (Related to Figure 2). A. Stable isogenic 293T-DPP4 cells were established and DPP4 expression level was measured using Alexa Fluor 488 conjugated anti-DPP4 antibody using flow cytometry. **B.** VLP entry data shown in **Figure 2B** (main Figure) were also collected upon increasing detection sensitivity in the luminescence plate reader. The data show that VSV-G VLPs have strong entry efficiency, although it is lower than SARS2 or SARS VLPs for 293T-hACE2 and A549-hACE2-TMPRSS2 cells. **C-D.** VSV-G and MERS spike plasmid mass was varied during production of 3P Luc-PS9 VLPs bearing these glycoproteins in 150mm petri dishes. VLPs produced using 4 µg VSV-G or 2 µg MERS spike plasmid caused maximal infection of target cells. **E.** Representative flow cytometry histograms for **Figure 2C** (main Figure) , showing that VLP tropism can be varied by changing viral glycoprotein**.** Data are Mean $\pm$ STD. *$P < 0.05$, **$P < 0.01$, ***$P < 0.001$, ****$P < 0.0001$, NS: not significant.

Figure S3
Yang et al.

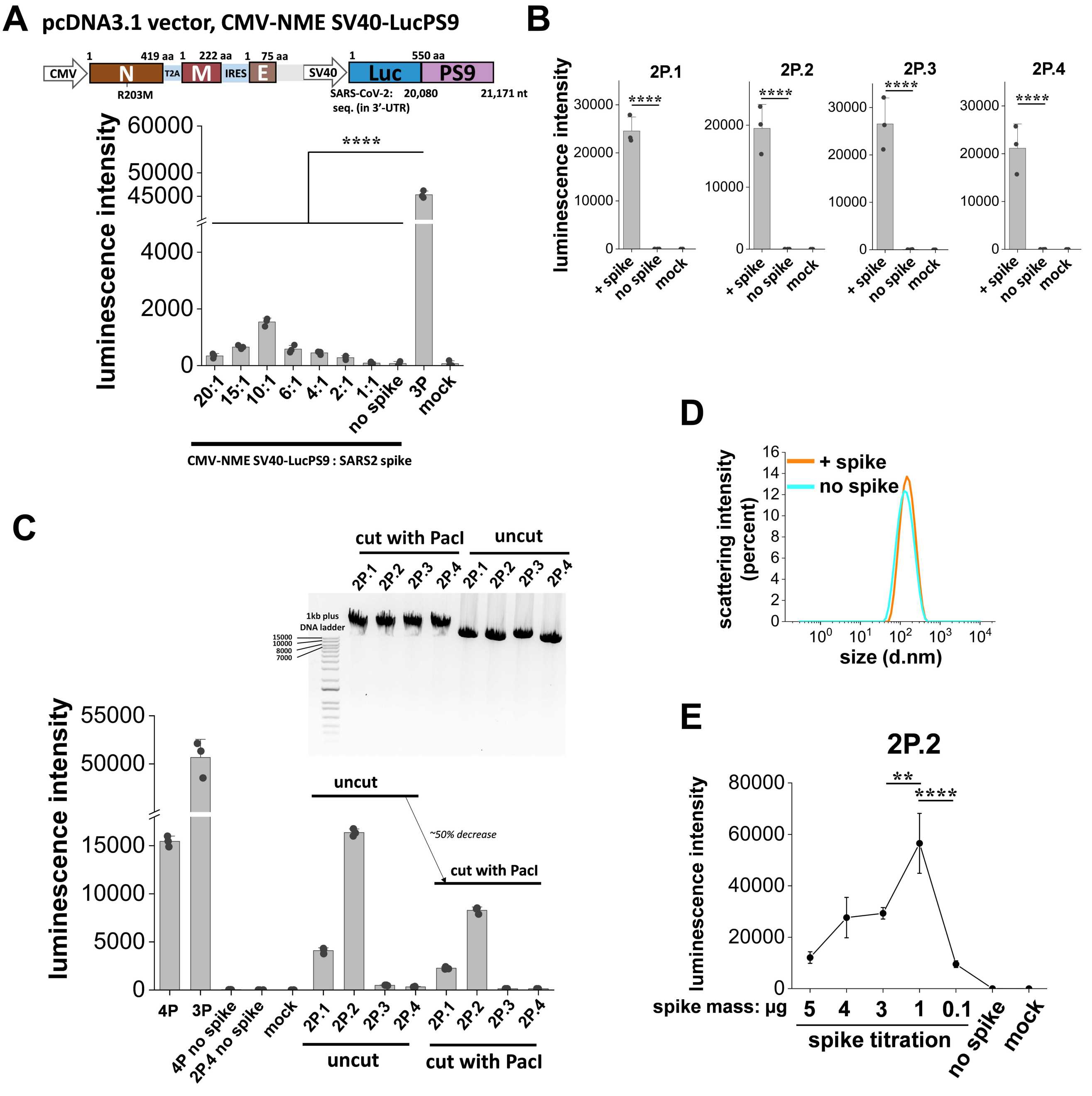

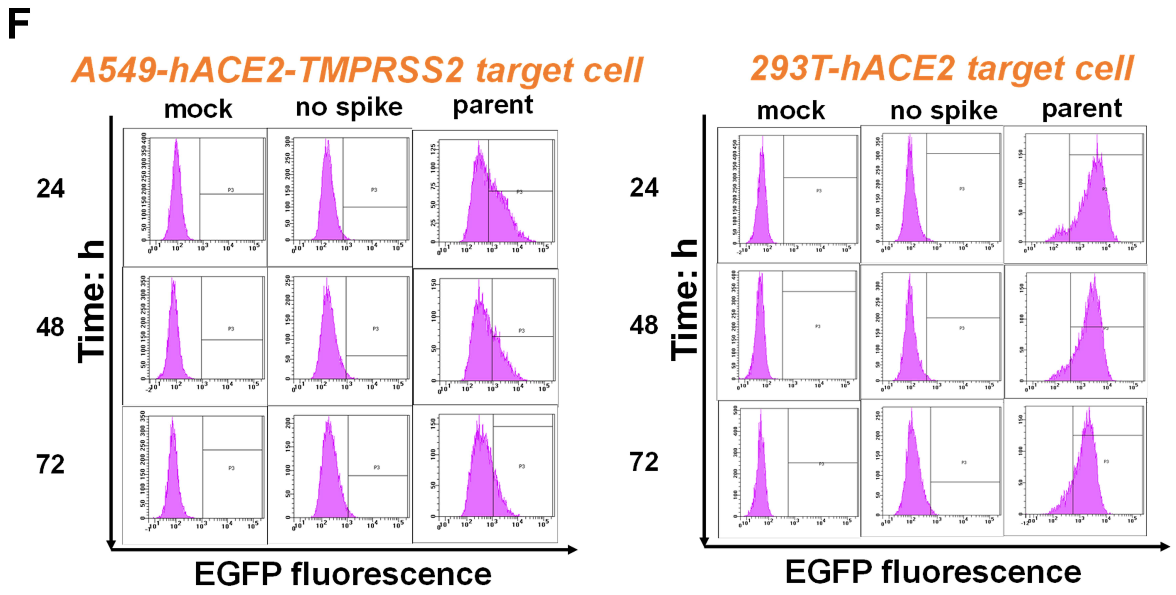

Figure S3. Characterization of 2P VLPs (Related to Figure 3). A. The NeoR/KanR resistance gene in pcDNA3.1 N-T2A-M-IRES-E plasmid, which is driven by the SV40 promoter was replaced by Luc-PS9. This new '2P' construct is called CMV-NME SV40-LucPS9. The proportion of this plasmid and SARS2 spike plasmid was varied during the production of SARS2 Luc-PS9 VLPs. Luminescence intensity in 293T-hACE2 cells was low for these particles in comparison to 3P VLPs, regardless of the VLP production condition. **B.** All four 2P SARS2 Luc-PS9 VLPs entered 293T-hACE2 cells in a spike dependent manner, albeit with different efficiencies. **C.** A restriction enzyme site *PacI*, embedded in the insulator/terminator region of 2P, was digested to linearize 2P.1, 2P.2, 2P.3 and 2P.4 during the VLP production step. Such cutting, which presumably eliminates any promoter interference, failed to improve the entry infectivity of the resulting VLPs. **D.** Dynamic light scattering (DLS) showing the particle size distribution of 2P.2 SARS2 Luc-PS9 VLPs. VLPs with spike (~146 nm) was sized slightly larger than the VLPs without spike (~125 nm). **E.** 2P.2 VLP production was optimal upon using 1 µg plasmid mass. **F.** Representative flow cytometry histogram data for **Figure 3E** (main Figure) showing appreciable VLP entry into hACE2 bearing cells when using 2P VLPs. Data are Mean ± STD. $^{**}P < 0.01$, $^{****}P < 0.0001$.

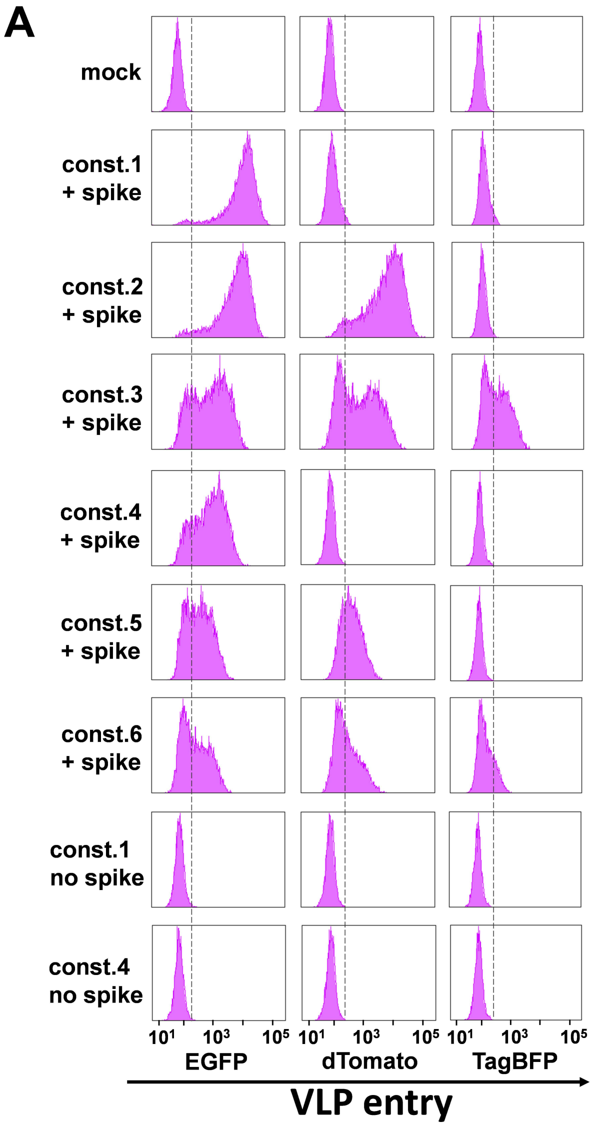

Figure S4. Effect of payload size on delivery (Related to Figure 4). A. Representative flow cytometry histograms for experiment in **Figure 4B** that quantified EGFP, dTomato and TagBFP fluorescence in 293T-hACE2 cells following addition of 3P SARS2 VLPs with different mRNA payloads. Data are presented for 'mock' and 'no spike VLPs' to illustrate background fluorescence and spike-dependent VLP entry. Vertical line separates background signal from signal due to spike dependent VLP entry.

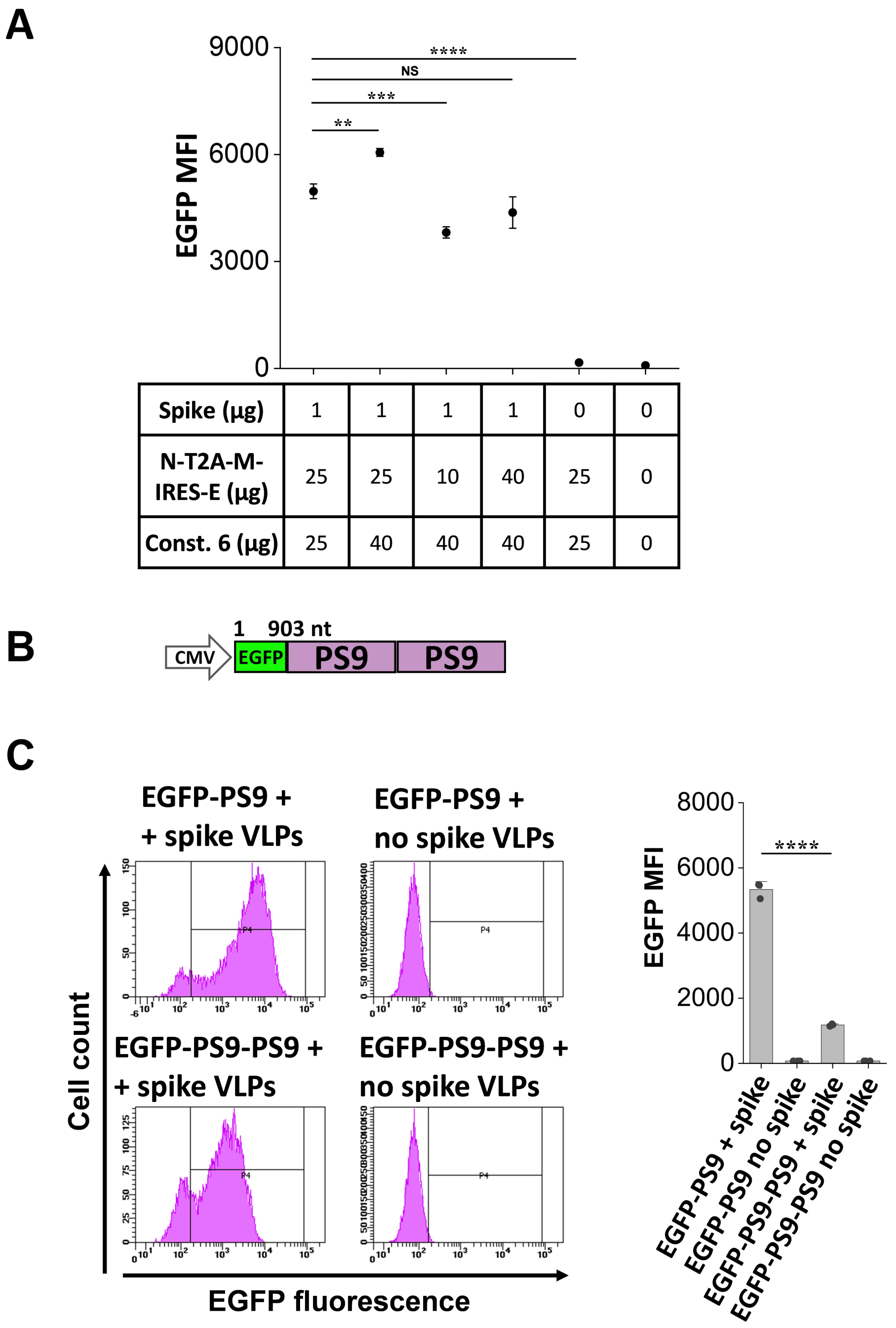

Spike (µg)	1	1	1	1	0	0
N-T2A-M-IRES-E (µg)	25	25	10	40	25	0
Const. 6 (µg)	25	40	40	40	25	0

Figure S5. Strategies to enhance VLP efficacy (Related to Figure 4). A. Plasmid amounts were varied during VLP production to determine optimal stoichiometry needed to generate 3P VLPs. These studies were performed using const. 6 as payload. Amounts of spike, N-T2A-M-IRES-E and const. 6 plasmid in each case are presented. Increasing const. 6 plasmid amount to 40 µg increased viral entry by ~25% compared to the standard stoichiometry that used equal amounts of N-T2A-M-IRES-E and const 6, both at 25 µg. Other conditions tested decreased EGFP signal. **B.** A duplicate PS9 packaging signal was appended at the end of the original PS9, resulting in an EGFP-PS9-PS9 construct. **C.** This construct failed to enhance viral entry measured based on fluorescence intensity for the 3P VLP system. Data are Mean ± STD. **$P < 0.01$, ***$P < 0.001$, ****$P < 0.0001$.

Figure S6
Yang et al.

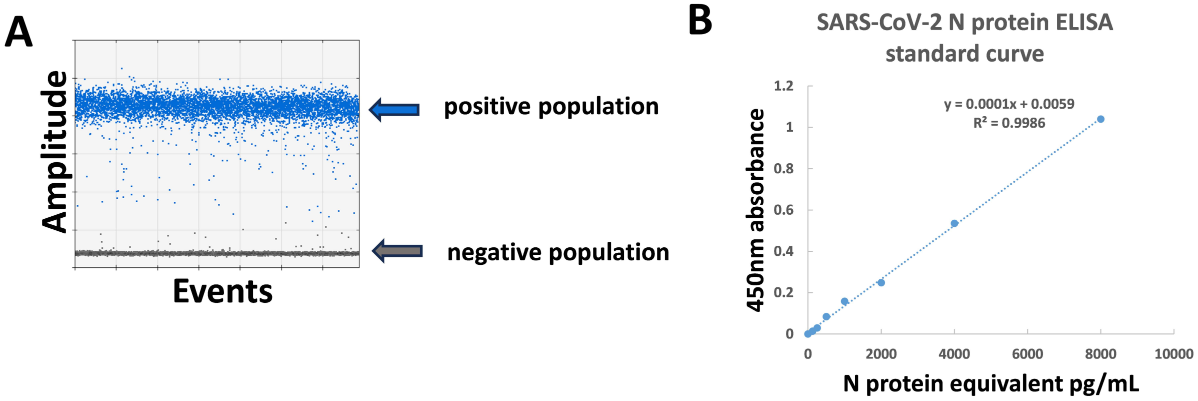

Figure S6. Characterization of VLPs using ddPCR and ELISA (Related to Figure 5). A. Representative ddPCR plot showing positive and negative population, when using probe directed against the PS9 sequence of the package. **B.** N protein ELISA standard curve for quantification of VLP concentration. VLP concentration was determined based on equivalent N protein amount incorporated into the particle.

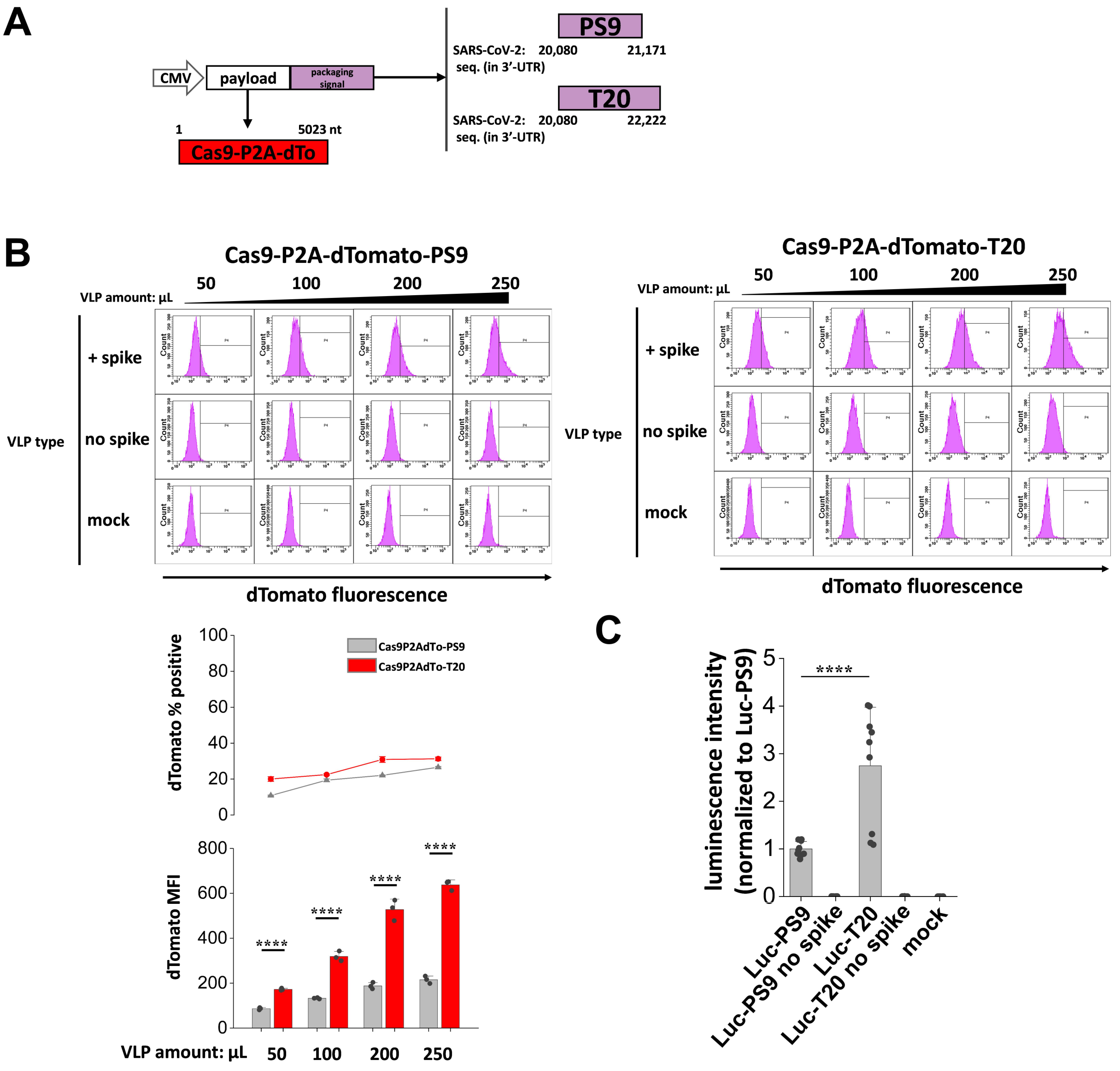

Figure S7. T20 is a more efficient packaging sequence compared to PS9 in the 3P VLP system (Related to Figure 6). A. 3P SARS2 Cas9-P2A-dTo VLPs (dTo: dTomato) were produced when the packaging signal was either PS9 or T20. **B.** VLP entry was measured in 293T-hACE2 cells based on the dTomato signal from either the 3P SARS2 Cas9-P2A-dTo-PS9 VLPs (left) or 3P SARS2 Cas9-P2A-dTo-T20 VLPs (right). VLP entry measured based on both % of cells that are dTomato positive and MFI was higher for 3P SARS2 Cas9-P2A-dTo-T20 VLPs compared to Cas9-P2A-dTo-PS9 VLPs across a range of VLP titers. **C.** Cas9-P2A-dTo-T20 was replaced by luciferase reporter in the above assay. Luminescence signal was ~2.5 times higher for 3P SARS2 Luc-T20 VLPs compared to 3P SARS2 Luc-PS9 VLPs. Due to these observations, T20 was used as packaging sequence in **Figure 6** and **7** (main Figure**)**. Data are Mean ± STD. ****$P < 0.0001$.

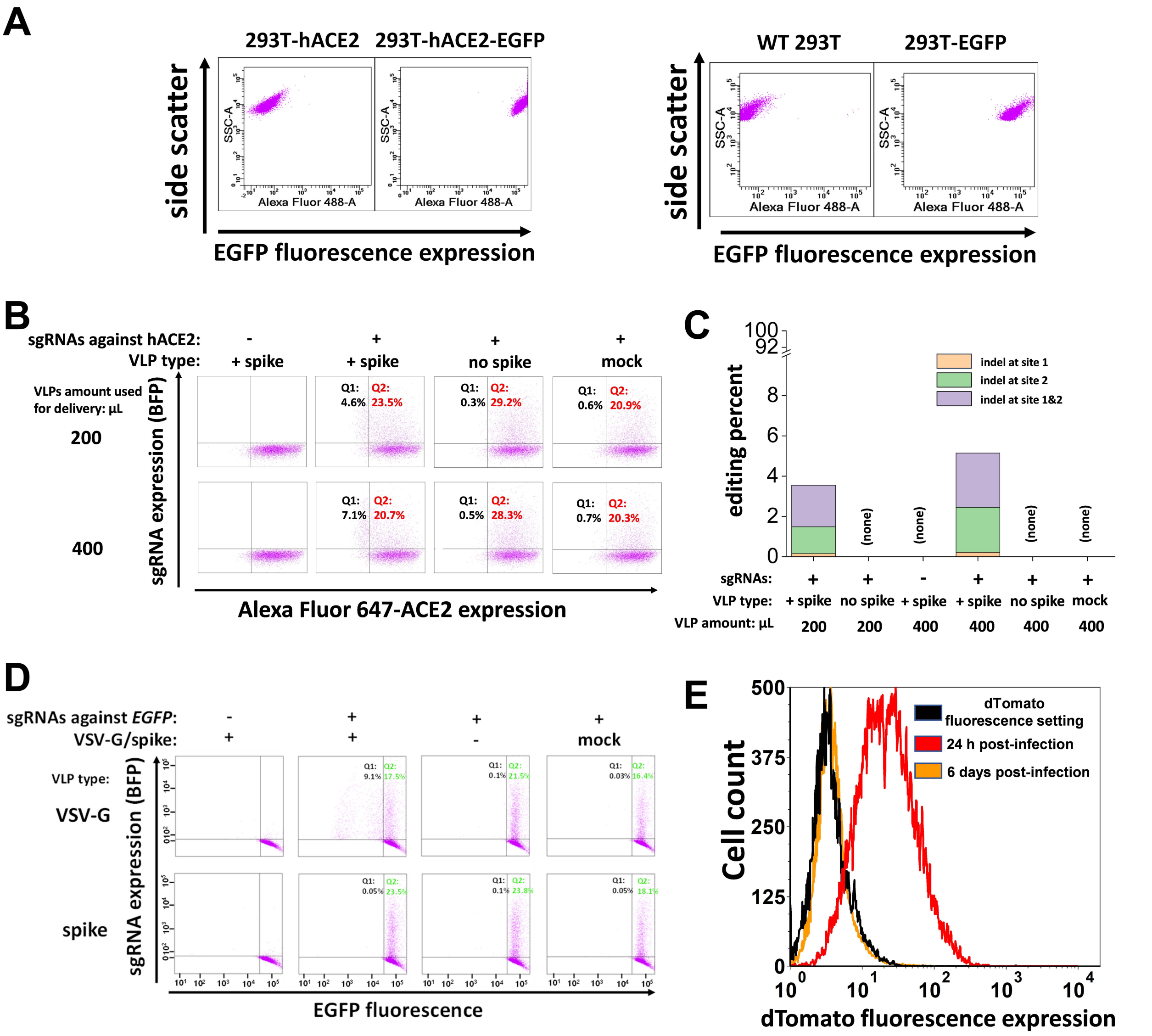

Figure S8. Gene editing of EGFP and human ACE2 (hACE2) (Related to Fig. 6). A. Stable isogenic 293T-hACE2-EGFP (left panel) and 293T-EGFP (right panel) cells were created for the spCas9 editing studies, by transduction using lentivirus carrying EGFP transgene. Both cell types expressed high levels of EGFP as assessed using flow cytometry. **B**. Representative dot plot of *hACE2* gene editing in 293T-hACE2 cells by SARS2 VLPs in **Figure 6D** (main manuscript). **C.** Genomic DNA was extracted from edited 293T-hACE2 cells in panel B studies. The edited region was PCR amplified and then sequenced using 150 bp paired end Illumina next-generation sequencing (NGS). Editing efficiency was quantified based on the site of editing in single reads (site 1, 2 or both sites) and this was normalized based on the total amplicon reads. **D.** Representative dot plot of gene editing of *EGFP* by VSV-G pseudotyped SARS-CoV-2 VLPs in **Figure 6E** (main manuscript). **E.** Histogram shows dTomato fluorescence intensity measured upon infection of 3P SARS2 Cas9-P2A-dTo-T20 VLPs into 293T-hACE2 cells at 24h and 6 days post-infection. A majority of cells were dTomato positive at 24h. Signal was absent 6 days post-infection, indicating the transient mRNA delivery into target cells.

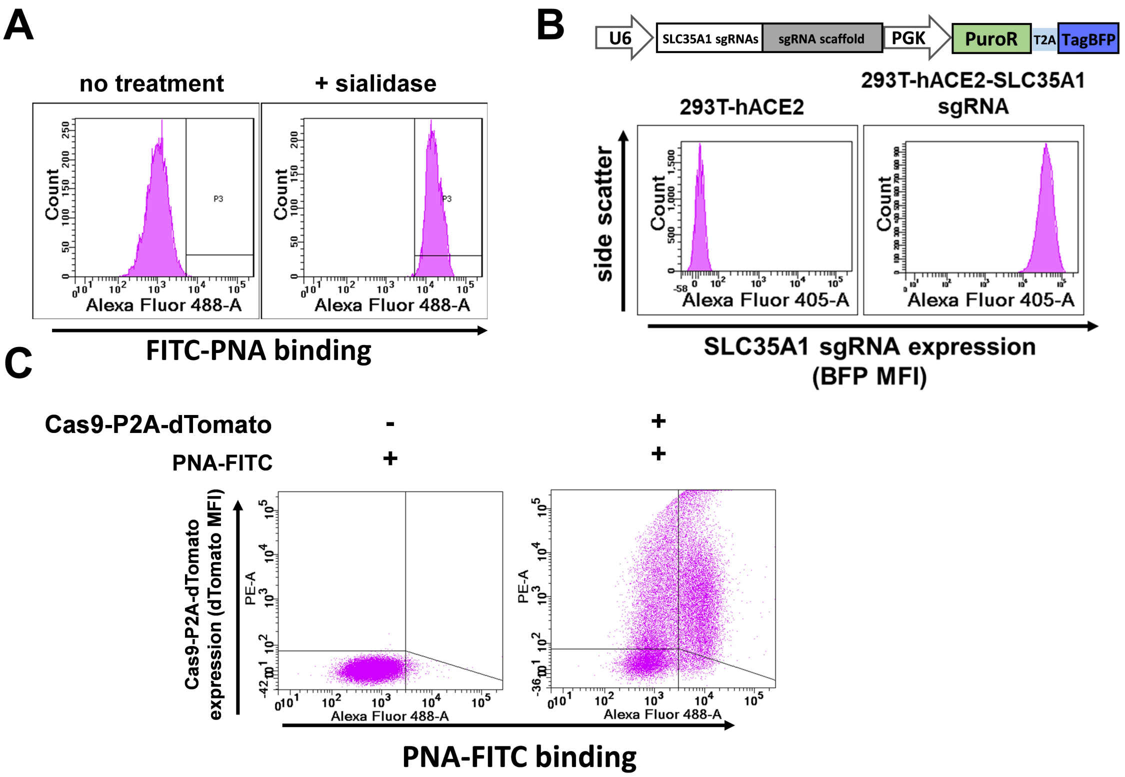

Figure S9. Knocking out *SLC35A1* in 293T-hACE2 cells augments peanut agglutinin lectin (PNA) binding (Related to Figure 6). A. 293T cells showed enhanced PNA binding following sialidase treatment. **B.** 293T-hACE2 cells stably express sgRNAs against *SLC35A1* (denoted as '293T-hACE2-SLC35A1 sgRNA' cells) were generated by transducing 293T-hACE2 cells with a pool of two VSV-G pseudotyped lentivirus each carrying an sgRNA targeting *SLC35A1*. Transduced cells were sorted based on BFP fluorescence (see vector schematci). **C.** 293T-hACE2-SLC35A1 sgRNA cells were transfected with Cas9-P2A-dTo-T20 plasmid. Editing efficiency [=100 × Top right / (Top right + Top left)] was ~65% in Cas9-P2A-dTomato positive cells at day 6, based on increased PNA binding. Representative cytometry plots are presented.

A

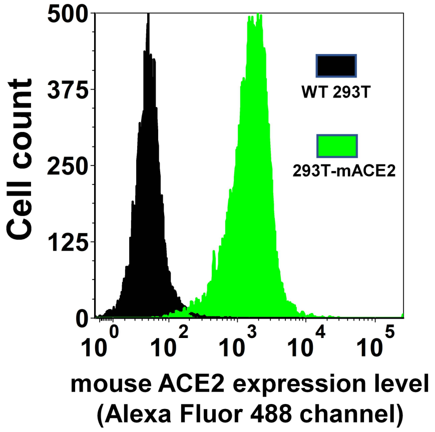

Figure S10. 293T mouse ACE2 ('293T-mACE2') cells (Related to Figure 7). A. 293T-mACE2 cells were produced by transducing VSV-G pseudotyped lentivirus carrying mouse ACE2 transgene into wild-type 293T cells. Mouse-ACE2 expressing cells were sorted using FACS and expression level quantified using anti-mouse ACE2 monoclonal antibody in flow cytometry run.
